# Host-guest assembly for highly sensitive probing of a chiral mono-alcohol with a zinc trisporphyrinate

**DOI:** 10.1038/s41598-017-03441-1

**Published:** 2017-06-19

**Authors:** Cong-Cong Zhuo, Li Li, Chuan-Jiang Hu, Jian-Ping Lang

**Affiliations:** 1 State and Local Joint Engineering Laboratory for Novel Functional Polymeric Materials, College of Chemistry, Chemical Engineering and Materials Science, Soochow University, Suzhou, 215123 Jiangsu P.R. China; 2 Applied Technology College of Soochow University, Suzhou, 215325 Jiangsu P.R. China

## Abstract

A zinc trisporphyrinate has been developed as a chirality sensor for chiral mono-alcohols. In its structure, there are two “spaces” surrounded by three porphyrin moieties, which allow guests to fill in. It has shown extremely high CD sensitivity for a chiral mono-alcohol with a naphthyl substituent, 1-(1-naphthyl)ethanol, at μM level, which is at least three orders of magnitude lower concentration than previous reports. A crystallographic study of the host-guest complex reveals the binding of 1-(1-naphthyl)ethanol to the zinc trisporphyrinate is greatly enhanced by multipoint interactions, such as coordination interactions, hydrogen bonding, π-π and CH···π interactions etc. Spectroscopic studies suggest the corresponding binding constant K_1_ is over 10^5^ M^−1^, which is two or three orders of magnitude larger than other mono-alcohols. Among porphyrin systems, this trisporphyrin have the strongest binding affinity for 1-(1-naphthyl)ethanol, which leads to the highest CD sensitivity.

## Introduction

Chiral alcohols are commonly encountered structural elements in pharmaceutical drugs, biologically active natural products and synthetic intermediate^1–3^. Determination of their absolute configurations is crucial to understand their functions, and the rapid determination of absolute configuration and enantiomeric excess for chiral molecules are also important for high-throughput screening of chiral catalysts^4^. Among these alcohols, mono-alcohols are more difficult to investigate due to their limited binding site and the weak binding affinity to metal centres. In past years, many efforts have been devoted to developing highly sensitive systems for recognition of chiral mono-alcohols. Some of them require the mono-alcohols to be derivatized^5–7^. Obviously, without chemical derivation, the determination of absolute configurations will be more convenient and rapid, especially through CD measurements.

Because of the intensive absorption in the Soret band, metalloporphyrins have been widely studied as good candidates for chirality related research^8–11^. However, because of the weak binding affinity of alcohols to metal centres, very few studies were reported^12–14^. For example, Borovkov and co-workers reported the ethane-bridged bisporphyrin system. In that case, the short ethane-bridge causes the steric interactions between the 3,7-ethyl groups of the porphyrin and the substituents of the ligand, resulting in the induced supramolecular chirality^12^. Another more sensitive system is an ester-linked bisporphyrin developed by Takanami *et al*., which improves the detecting concentration of mono-alcohols to mM level through the simultaneous double coordination of the hydroxyl group to the two metals^14^.

Although considerable progress has been made with respect to the recognition of chiral mono-alcohols, the search for highly sensitive sensors still remains challenge. In order to improve the sensitivity, one important strategy is to increase the host-guest interactions. In view of molecular recognition, such a purpose can be achieved through multipoint host-guest interactions.

**Figure 1:**
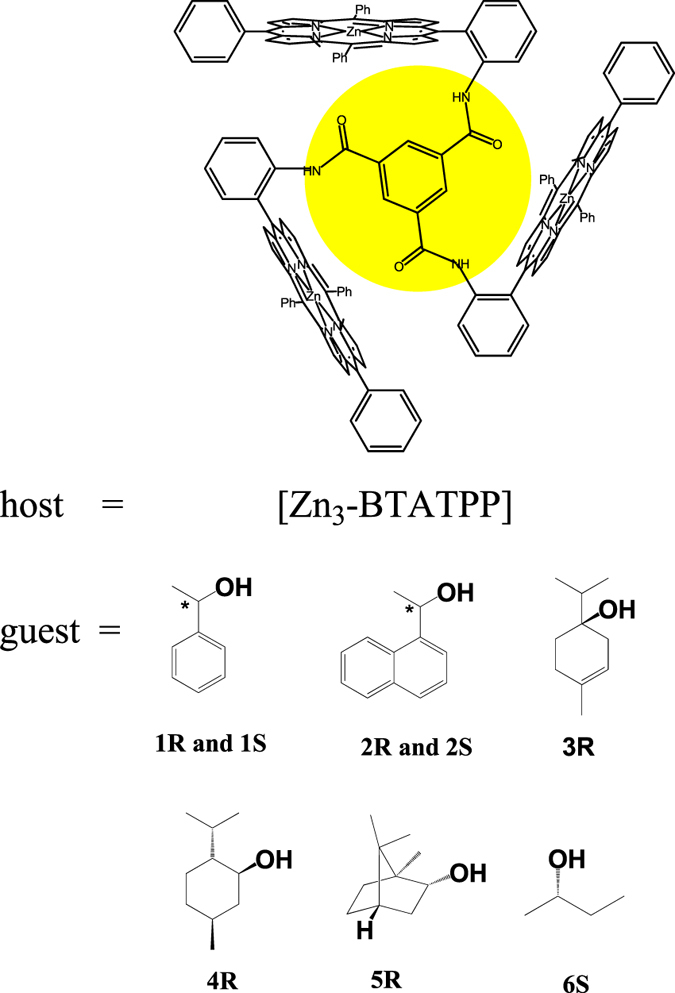
The structural formula of the host and guests. The host shows two “spaces” surrounded by three porphyrin moieties, one above and another below the linking phenyl plane.

We have recently reported a trisporphyrin system as shown in Fig. 1 ^15, 16^. In this trisporphyrin, the linking group is surrounded by three porphyrin moieties, which leads to the formation of two “spaces”, one above the linking phenyl and another below it. So this host has potential for chirality sensing of specific guests through multipoint interactions, such as coordination interactions, hydrogen bonding, π-π interactions and CH···π interactions etc., which will enhance host-guest interactions, and the disadvantage of mono-alcohols can be overcome.

In our previous study^16^, the crystal structure suggests the “space” formed by porphyrin moieties is a little big for 1-phenylethanol, and that “space” can accommodate one 1-phenylethanol and one H_2_O. A bulkier guest could match the “space” better. Herein, we examined several mono-alcohols. This Zn trisporphyrinate showed the highest sensitivity to 1-(1-naphthyl)ethanol (the guest **2**), the one with a naphthyl substituent. To obtain good CD signals, the concentration of the guest **2** only need to be at μM level, which is at least three orders of magnitude smaller than previous reports. To our knowledge, this is the most CD sensitive porphyrin system for chiral mono-alcohols so far.

## Results and Discussion

### Circular dichroism spectra

**Figure 2:**
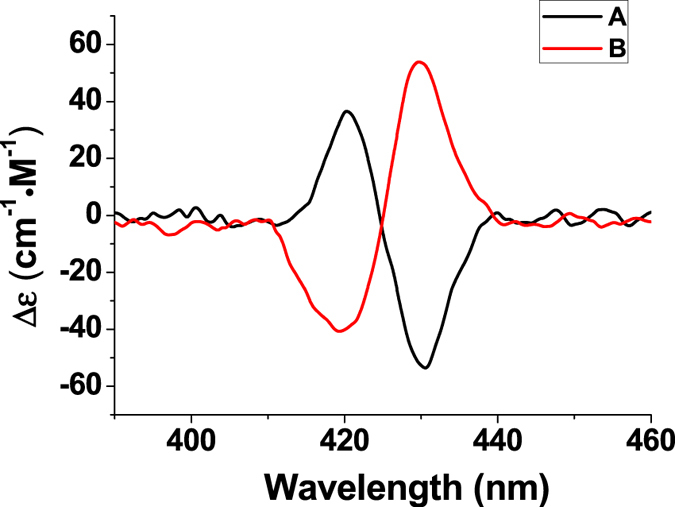
Circular dichroism spectra of a solution of [Zn_3_-BTATPP] (1.3 × 10^−6^ M) and 5.5 × 10^−7^ M of (**A**) S−1-(1-naphthyl)ethanol (**2S**), or (**B**) R-1-(1-naphthyl)ethanol (**2R**) in the mixture of hexane and methylene chloride (9:1) at 295 K.

The CD spectra for the mixture of [Zn_3_-BTATPP] and the guest **2** were shown in Fig. 2. When the concentration of guest **2** is 0.55 µM, there are remarkable bisignate CD signals in the Soret band region. As shown in Figure S5, when its concentration is 2.1 × 10^−5^ M, the A value reaches 1054 cm^−1^M^−1^, the strongest CD value in the reported porphyrin systems for mono-alcohols. For **2S**, the longer-wavelength peak of the Soret band was negative and the shorter-wavelength peak was positive. For **2R**, the CD spectra showed similar shapes and intensities but opposite signs in the Soret band region. So this host can be used to determine the absolute configuration of 1-(1-naphthyl)ethanol. The spectra for other mono-alcohols are shown in the supporting information. The corresponding guest concentrations at which the A_obs_ values are about 100 cm^−1^M^−1^ are also listed in Table 1. For the guest **2**, the detecting concentration is three orders of magnitude lower than those reported by Takanami *et al*.^14^. We also noticed there were limitations for this trisporphyrin system. For example, the signs of these CD spectra for these mono-alcohols did not show clear patterns as literatures reported. And this system is only highly sensitive to the specific case of 1-(1-naphthyl)ethanol. Except the guest **2**, the required concentrations for other guests are much higher, ranging from 10^−3^ to 10^−4^ M, which are similar to Takanami’s results.

**Table 1:** Observed CD spectral data of chiral mono-alcohols with the host [Zn_3_-BTATPP] (1.3 × 10^−6^ M) at 295 K

alcohol	[Zn_3_-BTATPP]		A_obs_^a^	C^b^ (cm^−1^M^−1^)
	Δε,	λ nm		
1R^c^	+55,	433	+97	7.3 × 10^−4^
	−42,	419		
1S^c^	−55,	433	−97	7.3 × 10^−4^
	+42,	419		
2R	+52,	430	+89	5.5 × 10^−7^
	−37,	420		
2S	−52,	430	−89	5.5 × 10^−7^
	+37,	420		
3R	−45,	428	−97	4.8 × 10^−3^
	+52,	420		
4R	−49,	432	−101	1.5 × 10^−4^
	+52,	424		
5R	+60,	431	+101	1.9 × 10^−4^
	−41,	419		
6S	+47,	422	+97	3.0 × 10^−3^
	−50,	416		

^a^A_obs_ = Δε_1_–Δε_2_, cm^−1^M^−1^. This value represents the total amplitude of the experimentally observed CD couplets. ^b^The concentration of the guest for the corresponding A_obs_. Ref. 16. All chiral reagent ee > 95%.

### Rationalization of the CD sensitivity for the guest 2

What causes that the CD sensitivity for the guest **2** is significantly higher than other mono-alcohols? The magnitude of CD signals relies on the following two factors: 1) The binding affinity of guests to hosts. It determines the amount of host-guest complexes formed at low concentrations. 2) The CD magnitude of the corresponding host-guest complex *per* unit. In the following studies, we have evaluated the contributions from both factors.

**Figure 3:**
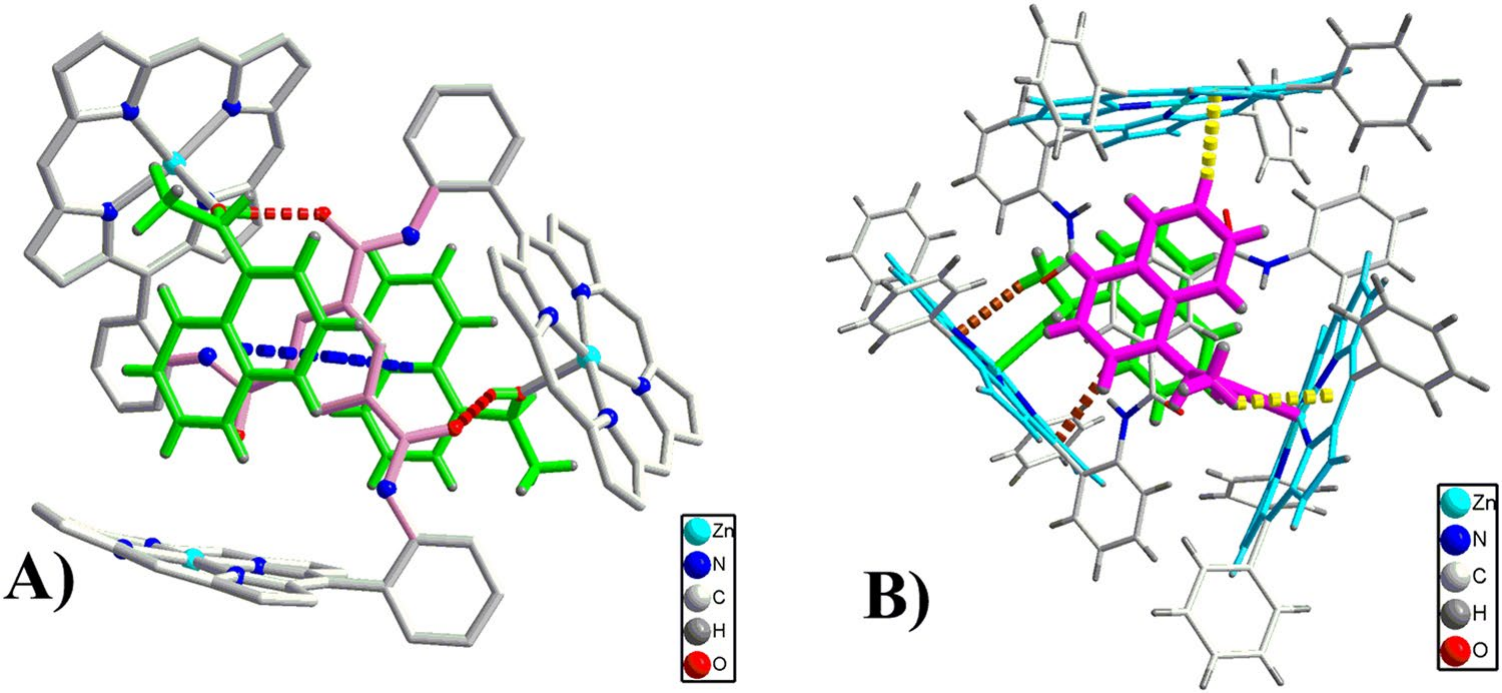
Crystal structure of [Zn_3_-BTATPP]·(**2S**)_2_. (mol A). (**A**) Showing hydrogen bonds (red dash line) and π-π interactions (blue dash line). Some phenyl groups at *meso*-positions and all hydrogen atoms except those for the guest are omitted for clarity. (**B**) Showing CH···π interactions (yellow dash line and brown dash line).

In order to evaluate the first factor, the binding affinity, we did studies on the crystal structure and the binding constants. When large excess of the guest **2** was mixed with the host, the suitable single crystals for the 1:2 host-guest complex were obtained. The structure was solved in *P*1, a chiral space group. In one asymmetric unit, there are two independent zinc trisporphyrinate molecules, mol A and mol B. The structure of mol A is displayed in Fig. 3, and the other is displayed in Figure S11. The main framework consists of three zinc porphyrinate subunits with a benzene tricarboxamide group as the linker. In each trisporphyrin, only two Zn centres are five-coordinate with **2S** as an axial ligand, while the third Zn is four-coordinate without any axial ligand. The overall formula is [Zn_3_-BTATPP]·(**2S**)_2_. Each **2S** adopts the “inside” binding mode. Besides the coordination interactions, hydrogen bonding and π-π interactions are also found between hosts and guests as shown in Fig. 3A. The carbonyl oxygens form hydrogen bonds with the coordinated OH, the corresponding O···O distances are 2.67 and 2.74 Å. The corresponding data are provided in Table S3. There are π-π interactions formed between the naphthyl rings of **2S** and the linking phenyl ring. The centroid-centroid distances are 3.55 Å and 3.72 Å, the dihedral angles are 2.0° and 8.4°, respectively. The two guests fit the two “spaces” surrounded by three porphyrin moieties to form a “sandwich” like structure.

As expected, 1-(1-naphthyl)ethanol is bulkier than 1-phenylethanol, which makes it match the “space” better, and also causes other interactions between hosts and guests. Besides coordination interactions, π-π interactions and hydrogen bonding interactions, there are also C-H···π interactions as shown in Fig. 3B. Obviously, the C-H···π interactions cause the blocking of the “inside” coordination position of the third zinc (the four-coordinate zinc), which leads to the 1:2 complex when the guests are in large excess.

The chirality of the crystal is confirmed by the solid CD spectrum in Figure S12, which shows clear signals in the Soret band region. The solid CD spectrum has a similar shape to the solution CD spectrum, which indicates the host-guest complex in solution could have a similar structure to that in the solid state. But we can not rule out other possibilities since intermolecular exciton couplings in the solid state could occur due to crystal packing^17, 18^. The crystal structure reveals there are multipoint interactions among the host and guests, which could increase the overall interactions, and thus increase the binding affinity. This is further confirmed by binding constant determination.

**Figure 4:**
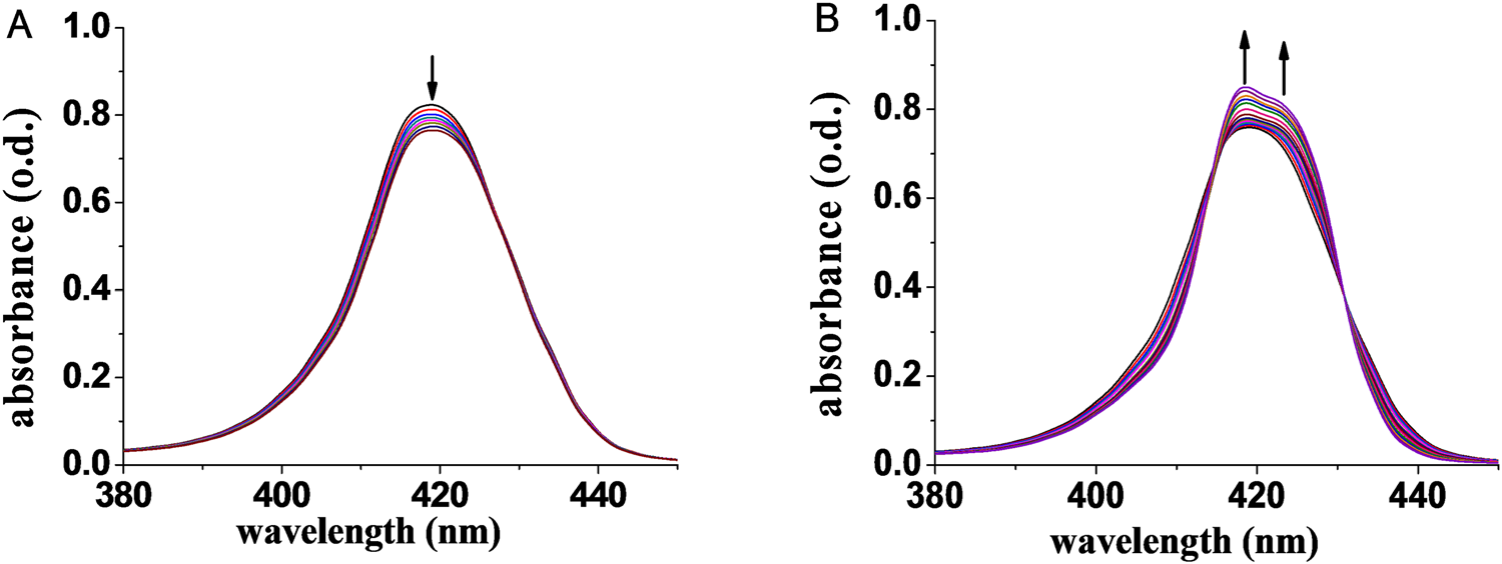
UV-vis spectral change of [Zn_3_-BTATPP] (1.2 × 10^−6^ M) upon addition of **2S** as its concentrations change from (**A**) 0–3.1 × 10^−6^ M, (**B**) 7.4 × 10^−6^–4.2 × 10^−4^ M.

The binding constants were determined from UV-vis titration spectra. As shown in Fig. 4, the UV-vis spectral change showed two steps: (1) When the concentrations of the guest **2** change from 0 to 3.1 × 10^−6^ M, the peak at 419 nm keep decreasing; (2) When the concentrations increase from 7.4 × 10^−6^ to 4.2 × 10^−4^ M, the peak at 419 nm starts to increase and a shoulder appears at 424 nm, then two isobestic points are observed at 415 and 431 nm. These spectral changes indicate there are the following two equilibriums in solution, where L present the guest. There could be three equilibriums since there are three Zn centres. But since the binding of the first and second guests blocks the “inside” coordination position of the third Zn, the third Zn has much weaker binding affinity than other two. Under our experimental conditions, the concentrations of the guest **2** is not high enough to cause the formation of the detectable amount of the 1:3 complex, and thus the third equilibrium can be ignored. Actually, the 1:2 complex in solution is confirmed by the Job’s plot based on the ^1^H NMR titrations at relative high concentrations (from 10^−3^ to 10^−2^ mol/L) (Figure S15). The peak at 0.33 mole fraction corresponds to a 1:2 host-guest complex.

1 $$[{{\rm{Zn}}}_{3}-\mathrm{BTATPP}]+{\rm{L}}\mathop{\rightleftharpoons }\limits^{{{\rm{K}}}_{1}}[{{\rm{Zn}}}_{3}-\mathrm{BTATPP}]\cdot {\rm{L}}$$

2 $$[{{\rm{Zn}}}_{3}-\mathrm{BTATPP}]\cdot {\rm{L}}+{\rm{L}}\mathop{\rightleftharpoons }\limits^{{{\rm{K}}}_{2}}[{{\rm{Zn}}}_{3}-\mathrm{BTATPP}]\cdot {(L)}_{{\rm{2}}}$$

The nonlinear least-squares program SQUAD^19^ was used to calculate the binding constants for the two equilibriums. More fitting details were provided in the supporting information (Figure S20). For **2S**, the binding constants K_1_ and K_2_ are found to be 1.8 (±0.1) × 10^5^ M^−1^ and 1.4 (±0.1) × 10^4^ M^−1^. The fitting of the CD spectral changes (Figs S25–S29) also gave these binding constants, which provided similar values (Table S6). For the guest **2**, K_1_ is the largest value in the reported porphyrin-monoalcohol complexes, which is 2 or 3 orders of magnitude larger than other mono-alcohols in Table 2, and two orders of magnitude larger than that in Takanami’s system^14^. The strong binding affinity to the guest **2** is consistent with the multipoint interactions in the structure.

**Table 2:** The binding constants between [Zn_3_-BTATPP] and mono-alcohols and calculated CD contributions *per* unit.

	K_1_ (M^−1^)	K_2_(M^−1^)	P1^a^ (%)	P2^b^ (%)	P1/P2	A_cal_^c^
1S	1.10(±0.06) × 10^3^	4.5(±0.5) × 10^2^	55	2.9	19	176
2S	1.8(±0.1) × 10^5^	1.4(±0.1) × 10^4^	7.1	0.04	178	1254
3R	1.8(±0.2) × 10^2^	1.6(±0.3) × 10	43.5	3.1	14	223
4R	2.8(±0.1) × 10^3^	9.9(±0.8) × 10^2^	29.0	4.5	6	348
5R	2.3(±0.3) × 10^3^	9.1(±1.3) × 10	30.4	0.54	56	332
6S	6.5(±0.6) × 10^2^	1.1(±0.1) × 10^2^	54.6	17.1	3	178

^a^P1, percentage of 1:1 complex at the corresponding low concentration C in Table 1; ^b^P2, percentage of 1:2 complex; ^c^A_cal_, calculated values by A_obs_/P1, A_obs_ is the value in Table 1.

The binding constant K_1_ values follow this order: **2** ≫ **5** **≈** **4** > **1** > **6** > **3**. For mono-alcohols **5**, **4**, **1** and **6**, this order is consistent with their bulkiness. For the case of **2**, its size is comparable with **5** (its overall volume is smaller than **5**, but its long axis (the longest distance between two atom) is actually longer than **5**). More importantly, the π-π or C-H···π interactions are absent for the guest **5**, which make its binding affinity much less than **2**. For the case of **3**, it is a tertiary alcohol, others are secondary alcohols, which probably causes its binding affinity much weaker than others.

The high binding constants for the guest **2** suggest that more host-guest complexes are formed than other mono-alcohols when their concentrations of guests (and hosts) are the same. To further evaluate the CD results, we have also to consider the second factor - the CD contribution of the corresponding host-guest complexes *per* unit.

We can actually obtain some direct information about CD contributions from experimental data. CD signals are contributed from both 1:1 and 1:2 host-guest complexes. For the CD sensitivity, we are concerned about the CD signals at low guest concentrations. Under such a condition, the amount of 1:1 complexes are much more than 1:2 complex as shown in Table 2. Thus the CD magnitude is mainly contributed from the 1:1 complexes, the CD magnitude *per* unit could be roughly treated as A_obs_/P1. As shown in Table 2, the CD contributions range from 176 to 1254 cm^−1^M^−1^ for different mono-alcohols. Obviously, among them, the 1:1 complex for the guest **2** leads to the largest CD magnitude *per* unit. So for the same amount of 1:1 complex, the CD amplitude for 1-(1-naphthyl)ethanol is several times (below 10) as other alcohols.

The above studies suggest that this zinc trisporphyrinate has the strongest binding affinity to 1-(1-naphthyl)ethanol and the host-guest complex has the largest CD magnitude *per* unit, which leads to the highest CD sensitivity. If we consider the binding constants for 1-(1-naphthyl)ethanol is over 100 times larger than others and its CD contribution *per* unit is only a few times than others, the major factor causing the highest CD sensitivity for the guest **2** should be the strong bonding affinity, which is due to the strong host-guest interactions.

## Conclusion

In conclusion, a zinc trisporphyrinate has shown high CD sensitivity at µM level for 1-(1-naphthyl)ethanol. The crystallographic study reveals that coordination interactions, hydrogen bonding, π-π and CH…π interactions in the host-guest complex cause strong host-guest interactions, which is confirmed by the large binding constant. Our studies suggest the “space” formed by porphyrin moieties is “recognized” best by the chiral mono-alcohol with the naphthyl substituent, the guest **2**, which is crucial to chiral recognition of mono-alcohols. We could adjust the substituents in hosts to create the right “space” for the specific guest, which can improve the corresponding recognition ability and sensitivity. This type of hosts can be used as powerful tools for molecular recognition of some specific chiral molecules, and have also potential applications in catalytic synthesis. Further studies on other guests and derived hosts are under investigation.

## Experimental section

### Material and Physical Methods

Reactions involving moisture sensitive reagents were carried out under a nitrogen atmosphere using standard vacuum line techniques and glassware that was flame-dried and cooled under nitrogen before use. CH_2_Cl_2_ and n-hexane were distilled over CaH_2_. Triethylamine (Et_3_N) was distilled over KOH and then used immediately. Zinc 5-(2-aminophenyl)-10,15,20-triphenyl-porphyrinate was prepared as described in the literature^20^. The porphyrin host [Zn_3_-BTATPP] was prepared according to the reported method^15^. Elemental analyses were performed on an Elementar Vario EL III analytical instrument. A Shimadzu UV-3150 spectrometer was used to record UV−vis spectra. A MT Model J-815 spectrophotometer was used to record CD spectra at 25 °C in 10% CH_2_Cl_2_/hexane (scan speed: 50 nm/min, date pitch: 0.5 nm, bandwidth: 2 nm, response time: 1 seconds).

### CD and UV-vis measurements

Solutions of various guests in 10% CH_2_Cl_2_/hexane were added to the [Zn_3_-BTATPP] solutions at 25 °C. Then CD and UV-vis spectra were recorded. The corresponding CD spectra were normalized based on the [Zn_3_-BTATPP] concentration.

According to the following method, the measurement of the solid CD spectrum was performed. A single crystal and some solid KBr were grinded under high pressure to get the KBr pellets (the thickness is 0.3 mm), and then CD spectrum was recorded.

### Job’s continuous plot analysis to determine complex stoichiometry

The complex stoichiometry for the host-guest complexes was determined through ^1^H-NMR titration of [Zn_3_-BTATPP] and the chiral guest 2R. Upon complexation of 2R with [Zn_3_-BTATPP]^1^,HNMR spectra undergo changes in chemical shift (Δδ) due to the anisotropic effect of the porphyrin rings. These changes can be used to determine the composition of host-guest complexation in solution. The ^1^H-NMR titration [Zn_3_-BTATPP] with 2R (Figure S14) demonstrates changes in the chemical shifts of the protons upon binding of the guest to the host.

For each titration, 4.7 mg of [Zn_3_-BTATPP] (2.1 × 10^−6^ mol) was dissolved in CDCl_3_ (0.50 mL), and the NMR spectrum was recorded (TMS was used as internal standard). Following this, 0.3, 0.6, 1.0, 1.2, … up to 20 equivalents of 2R was added to the above solution; the NMR spectra was recorded after each addition.

Job’s plot was obtained with respect to changes in chemical shift (Δδ) of one of the [Zn_3_-BTATPP] protons (peak at 3.25 ppm, Figure S14). The molar fraction of [Zn_3_-BTATPP] (X_p_) was multiplied by the changes in ^1^H-NMR chemical shift of [Zn_3_-BTATPP] (Δδ_p_) for each titration data point. The resulting value was plotted against the molar fraction of [Zn_3_-BTATPP]. Peaking at 0.33 mol fraction corresponds to a 1:2 host-guest complex (Figure S15).

### Preparation of crystals of [Zn_3_-BTATPP]·(2S)_2_

The solution of [Zn_3_-BTATPP] (10 mg, 0.004 mmol) in toluene (4 mL) was mixed with the solution of S-1-(1-naphthyl)ethanol (9.4 mg, 0.05 mmol) in CH_2_Cl_2_ (1 mL), and the mixture was stirred for 3 min, then transferred into glass tubes (8 mm × 250 mm). n-Heptane was added as the nonsolvent at room temperature. After one months, purple crystals were then isolated (5.8 mg, yield 51%). Anal. Calcd for C_330_H_222_N_30_O_10_Zn_6_: C, 76.92; H, 4.34; N, 8.10; Found: C, 74.89; H, 4.40; N, 8.83.

### X-ray structure determination

A Bruker APEX-II CCD was used for data collection at 293(2) K, which is equipped with graphite monochromated Mo Kα (λ = 0.71073 nm). Direct methods were used to solve the structure, further refinements were done on *F* ^*2*^ by full-matrix least-squares technique using the SHELXL-2014 program package^21^. In the asymmetric unit, there are two molecules of [Zn_3_-BTATPP]·(**2S**)_2_. Non-hydrogen atoms were refined anisotropically and all hydrogen atoms were included in calculated positions. In the structure, several highly disordered solvent molecules were present, which could not be modeled successfully and, therefore, SQUEEZE^22^ was used. The electron count within the interporphyrin voids was 86 e, which corresponds to one molecule of dichloromethane per [Zn_3_-BTATPP]·(**2S**)_2_. Data collection parameters are listed in Table S1. The CIF file for the crystal structures were deposited at the Cambridge Crystallographic Data Centre (CCDC). The CCDC deposition number is 1504576.

## Electronic supplementary material

Supporting information for Host-guest assembly for highly sensitive probing of a chiral mono-alcohol with a zinc trisporphyrinate
