# Supplementary material for: Host-guest assembly for highly sensitive probing of a chiral mono-alcohol with a zinc trisporphyrinate
Source: Sci Rep. 2017 Jun 19;7:3829. doi: 10.1038/s41598-017-03441-1 (PMC5476613; doi:10.1038/s41598-017-03441-1)
Supplement: Supplementary file 1 — Supporting information for Host-guest assembly for highly sensitive probing of a chiral mono-alcohol with a zinc trisporphyrinate [file 41598_2017_3441_MOESM1_ESM.pdf]

Supporting Information for

Host-guest assembly for highly sensitive probing of a chiral mono-alcohol with a zinc trisporphyrinate

Congcong Zhuo, Li Li, Chuanjiang Hu* and Jianping Lang*

UV-vis and CD fitting details…..……….…………………………………...……...S2

FiCD spectra of [Zn_3-BTATPP] by different alcohols when the CD strength is about 100 $cm^{-1}M^{-1}$ (Figures S1-S4) …..……….…………………………………...……...S3

CD titration spectra of [Zn_3-BTATPP] by different alcohols (Figures S5-S10) …..S5

Crystal structure showing hydrogen bonding, π-π and CH…π interactions in [Zn_3-BTATPP]·$(2S)_2$ (mol B)（Figure 11） ……………………….………….…S8

Solid CD spectrum of [Zn_3-BTATPP]·$(2S)_2$ (Figure S12) S8

^{1}H-^{1}H gCOSY spectrum of [Zn_3-1] (Figure S13) S9

^{1}H-NMR spectra of titration of [Zn_3-BTATPP] with 2R (Figure S14) S9

Job's continuous plot of [Zn_3-BTATPP] with 2R (Figure S15) S10

UV-vis spectra change of [Zn_3-BTATPP] by different alcohols (Figures S16-S19).S11

The fitting graphs for [Zn_3-BTATPP] and different alcohols by UV-vis spectra (Figures S20-S24)..S13

The fitting graphs for [Zn_3-BTATPP] and different alcohols by CD spectra (Figures S25-S29) S18

Crystal data and structural refinements of [Zn_3-BTATPP]·$(2S)_2$ (Table S1)..……..S23

Selected bond distances for [Zn_3-BTATPP]·$(2S)_2$ (Table S2) S24

Bond lengths (Å) and angles (°) for hydrogen bonds for [Zn_3-BTATPP]·$(2S)_2$ （Table S3）……...S24

Analysis of π···π interactions (Table S4) S25

Analysis of CH···π interactions (Table S5) S25

Calculated binding constants (Table S6) …..S26

UV-vis and CD Fitting details.

The nonlinear least-squares program SQUAD was used to calculate the binding constants for the two equilibriums. This program is used to calculate the best values for the stability constants of the proposed equilibrium model by employing a nonlinear least-squares approach. The program is completely general in scope, having the capability to refine stability constants for the general complex $M_mM_l'H_jL_nL_q'$, where *m, I, n, q* ≥ 0 and j is positive (protons), negative (hydroxide ions), or zero.

For each absorbance value *Ai •k* the equation

$$A_{i,k} = \sum_{1}^{J} [\text{species}]_{i,j} \times \epsilon_{j,k} \qquad (1)$$

where [species]i,j is the concentration of the jth species in the ith solution (spectrum) and *Ej,k* is the molar absorptivity of the jth species at the *kth* wavelength. SQUAD computes the values of the overall formation constant(s) which minimize the sum of the squared residentials between observed and calculated absorbance values:

$$U = \sum_{1}^{K} \sum_{1}^{I} (A_{i,k}^{\text{obs}} - A_{i,k}^{\text{calc}})^2 \qquad (2)$$

The main program starts by initializing I/O unit numbers and maximum sizes for all execution-time dimensioned arrays. Subroutine PREPRO is then called to perform the majority of the data-input and input file error checking.

Typically, in the initial input, we input the initial values for the each formation constants, UV-vis observed absorbance values, molar absorption for the host etc., then the fitting will give the final refined formation constants, calculated UV-vis absorption values, molar absorption for the host-guest complex, standard deviations, etc.

We run the fitting multiple times starting from different initial formation constants (ranging from 10 to 10^5), the fitting gave the same results. The fitting results were also summarized in Table S6.

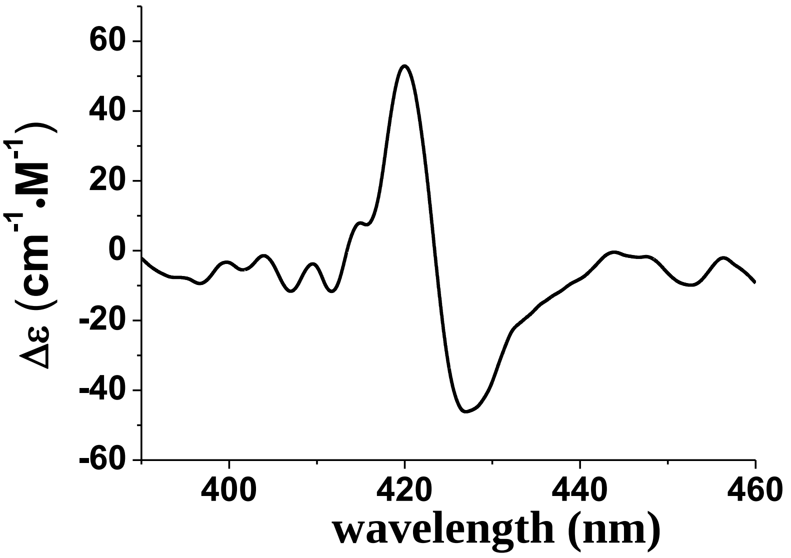

Figure S1. Circular dichroism spectrum of a solution of [Zn_3-BTATPP] (1.3×10^{-6} M) and 3R (4.8×10^{-3} M).

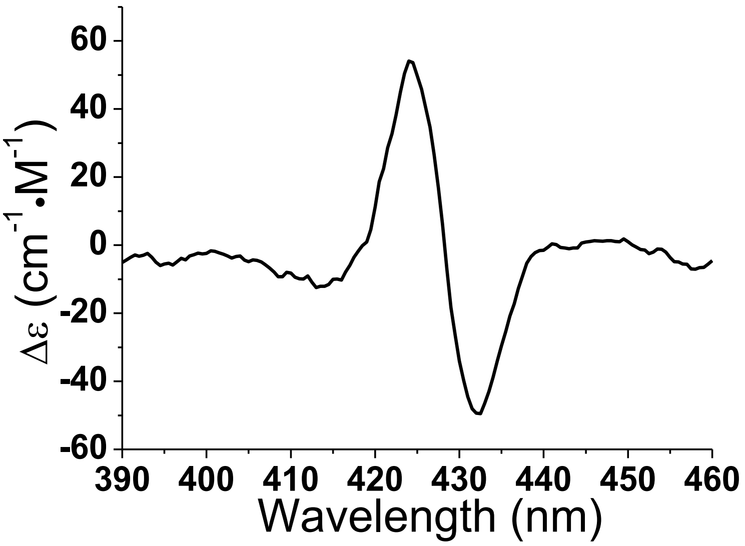

Figure S2. Circular dichroism spectrum of a solution of [Zn_3-BTATPP] (1.3×10^{-6} M) and 4R (1.5×10^{-4} M).

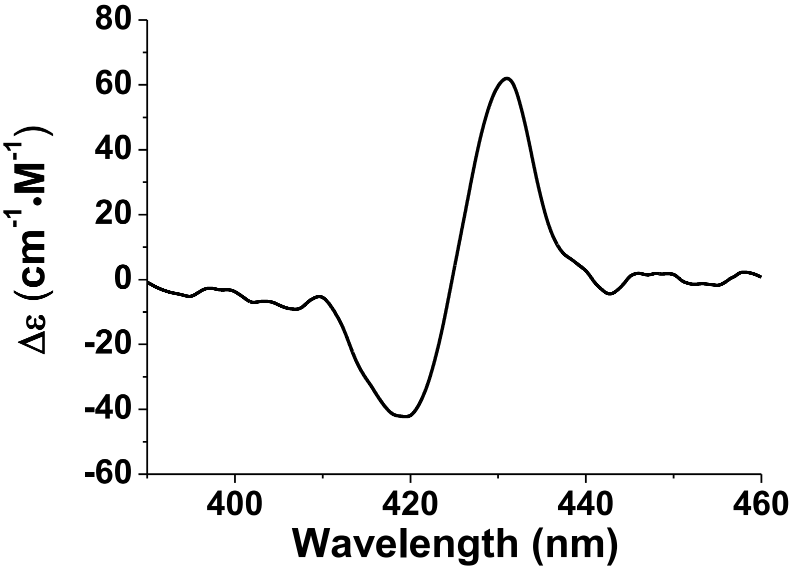

Figure S3. Circular dichroism spectrum of a solution of [Zn_3-BTATPP] (1.3×10^{-6} M) and 5R (1.9×10^{-4} M).

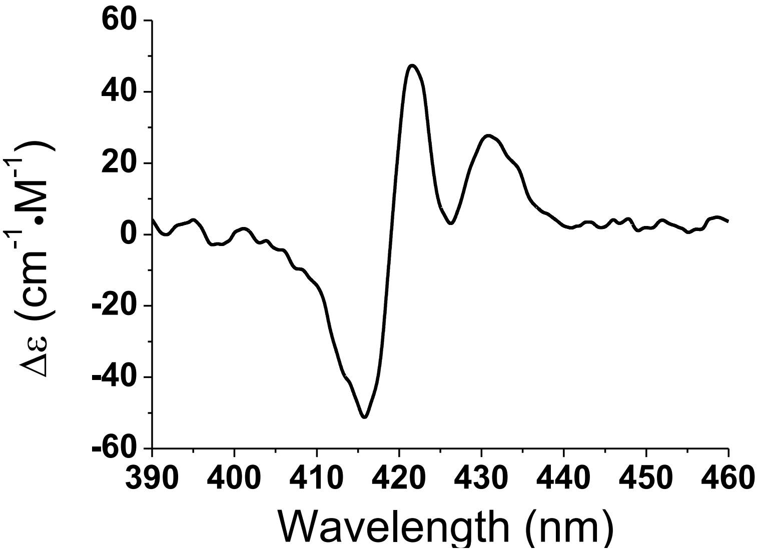

Figure S4. Circular dichroism spectrum of a solution of [Zn_3-BTATPP] (1.3×10^{-6} M) and 6S (3.0×10^{-3} M).

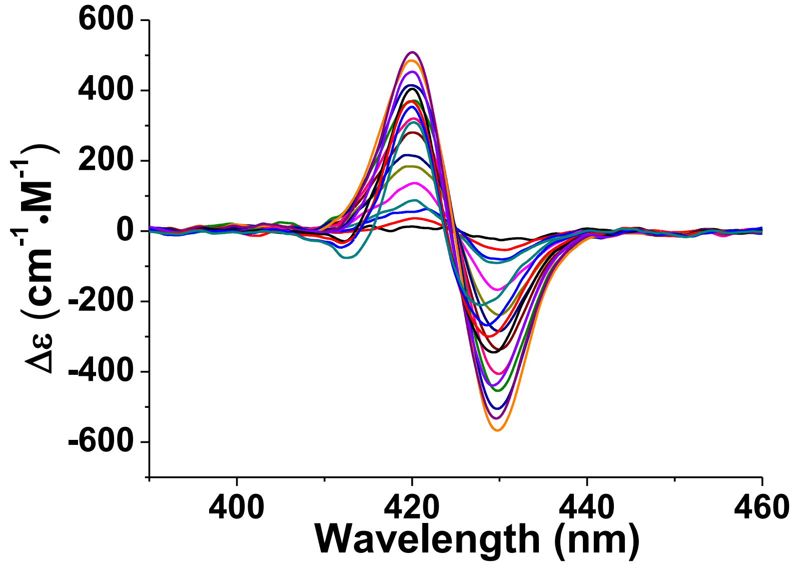

Figure S5. CD titration spectra of [Zn_3-BTATPP] (1.30×10^{-6} M) and 2S ($0\text{-}2.3\times10^{-3}$M).

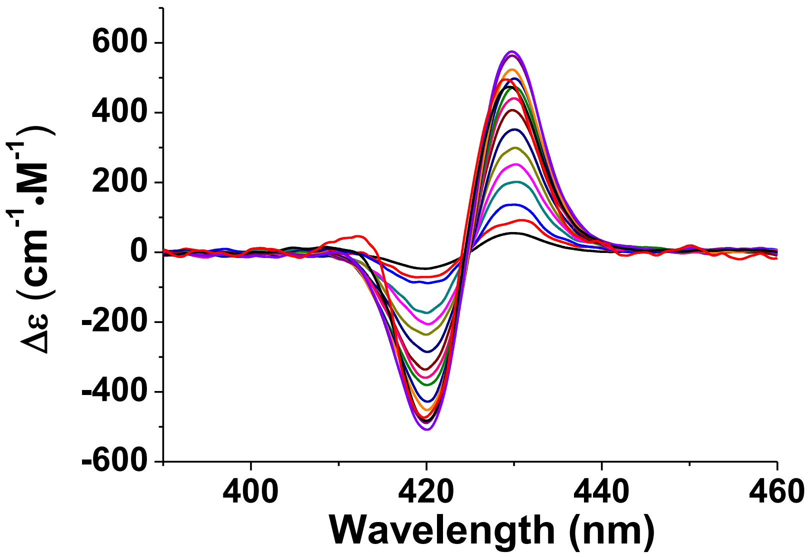

Figure S6. CD titration spectra of [Zn_3-BTATPP] (1.3×10^{-6} M) and 2R ($0\text{-}2.3 \times 10^{-3}$M).

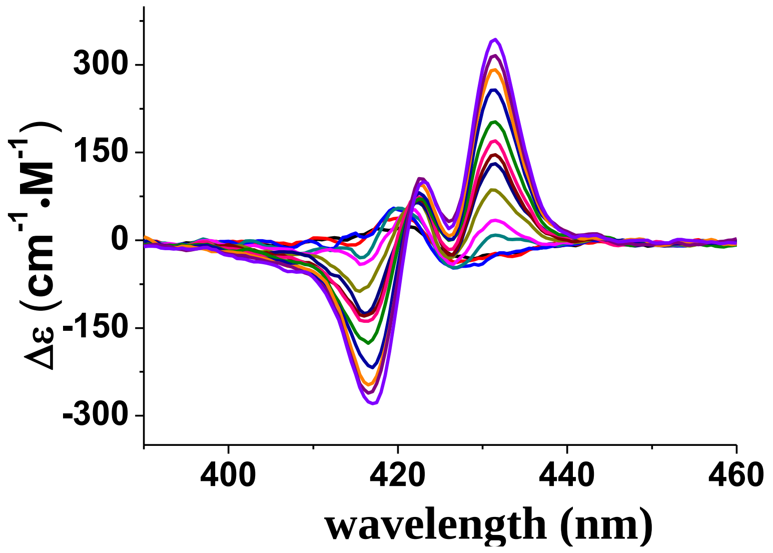

Figure S7. CD titration spectra of [Zn_3-BTATPP] (1.3×10^{-6} M) and 3R ($0\text{-}9.8 \times 10^{-2}$ M).

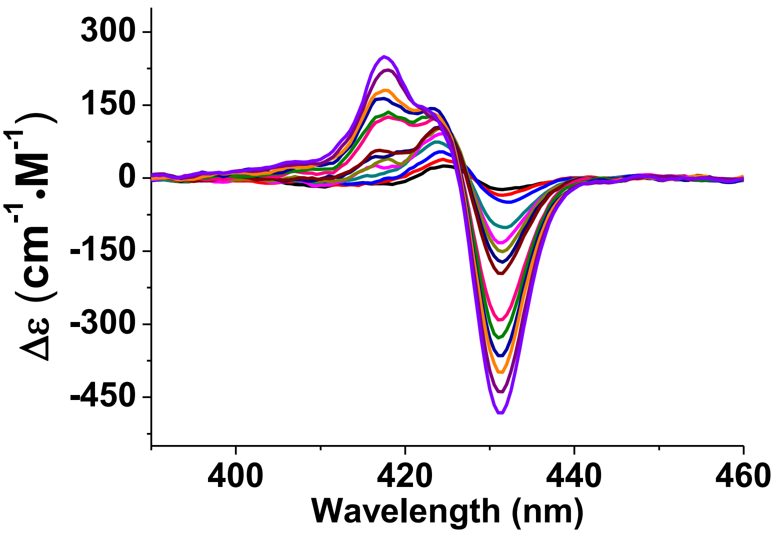

Figure S8. CD titration spectra of [Zn_3-BTATPP] (1.3×10^{-6} M) and 4R ($0\text{-}5.6 \times 10^{-3}$ M).

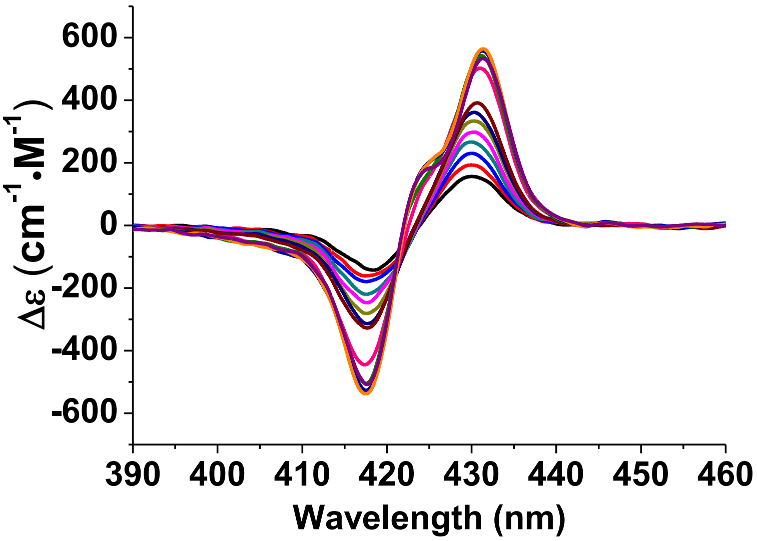

Figure S9. CD titration spectra of [Zn_3-BTATPP] (1.3×10^{-6} M) and 5R (0-1.2×10^{-1} M).

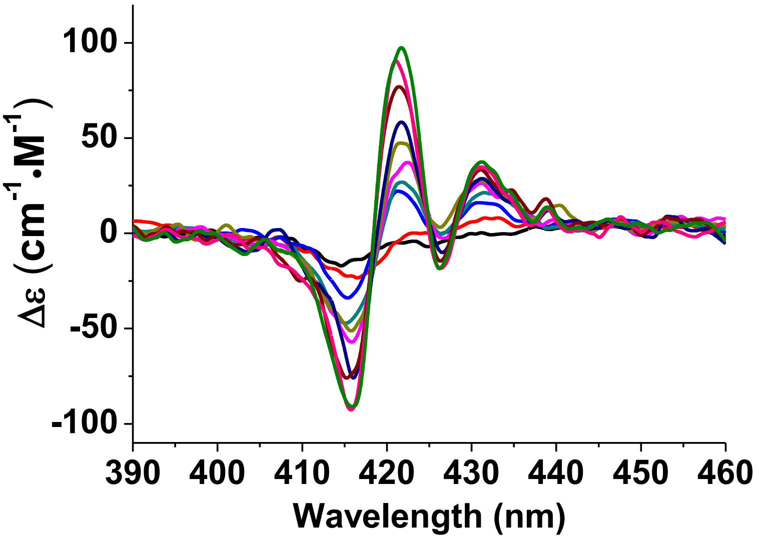

Figure S10. CD titration spectra of [Zn_3-BTATPP] (1.3×10^{-6} M) and 6S (0-7.6×10^{-3} M).

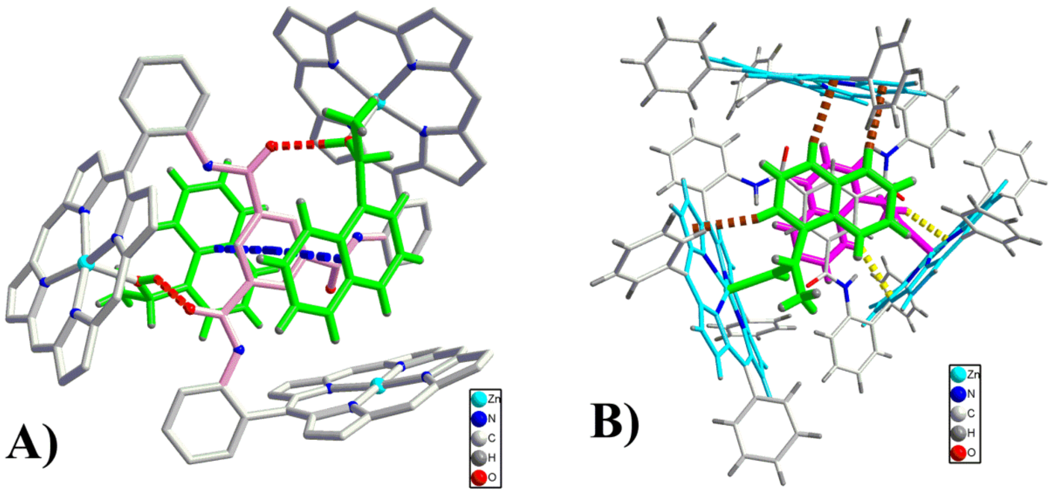

Figure S11. X-ray crystal structure of $[Zn_3\text{-BTATPP}]\cdot(\mathbf{2S})_2$. (mol B). A) Showing hydrogen bonds (red dash line) and π-π interactions (blue dash line). Some phenyl groups at *meso*-positions and all hydrogen atoms except those for the guest are omitted for clarity. B) Showing CH··· π interactions (yellow dash line and brown dash line).

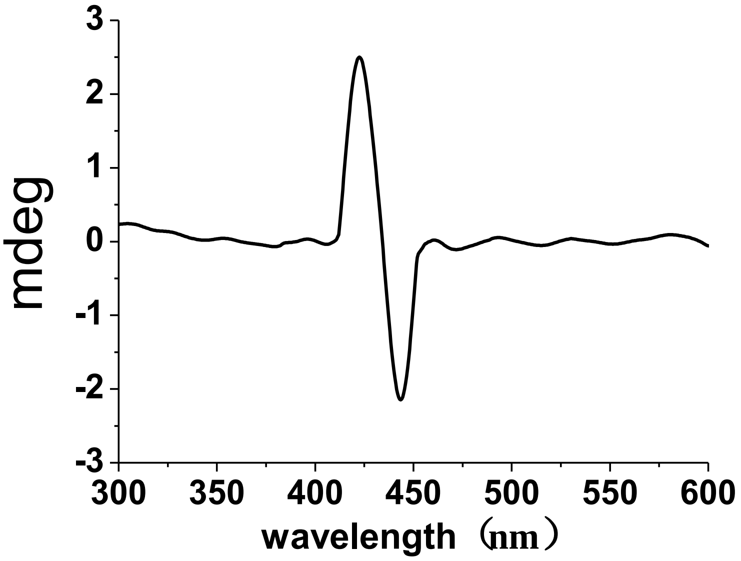

Figure S12. CD spectrum of a single crystal of $[Zn_3\text{-BTATPP}]\cdot(2S)_2$ in the KBr pellet.

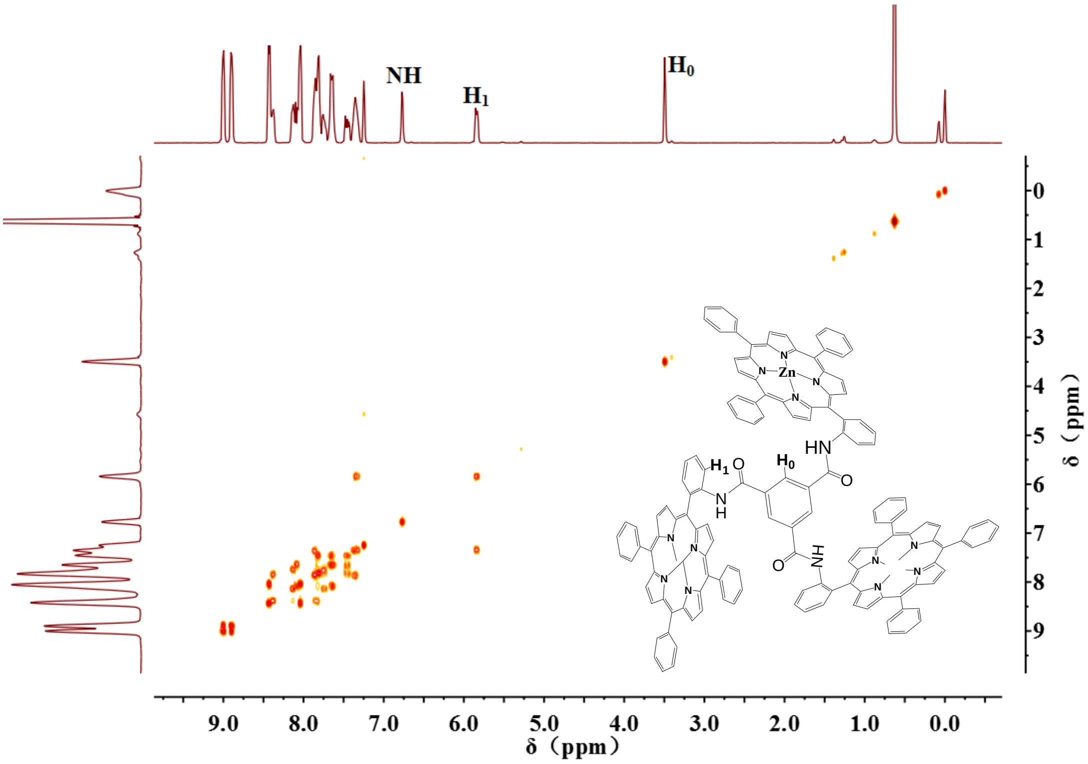

Figure S13. ^1H-^1H gCOSY spectrum of [Zn_3- BTATPP] (4.2×10^{-3} M) in $CDCl_3$ at 295 K. Inset shows the proton numbering scheme of [Zn_3-BTATPP]

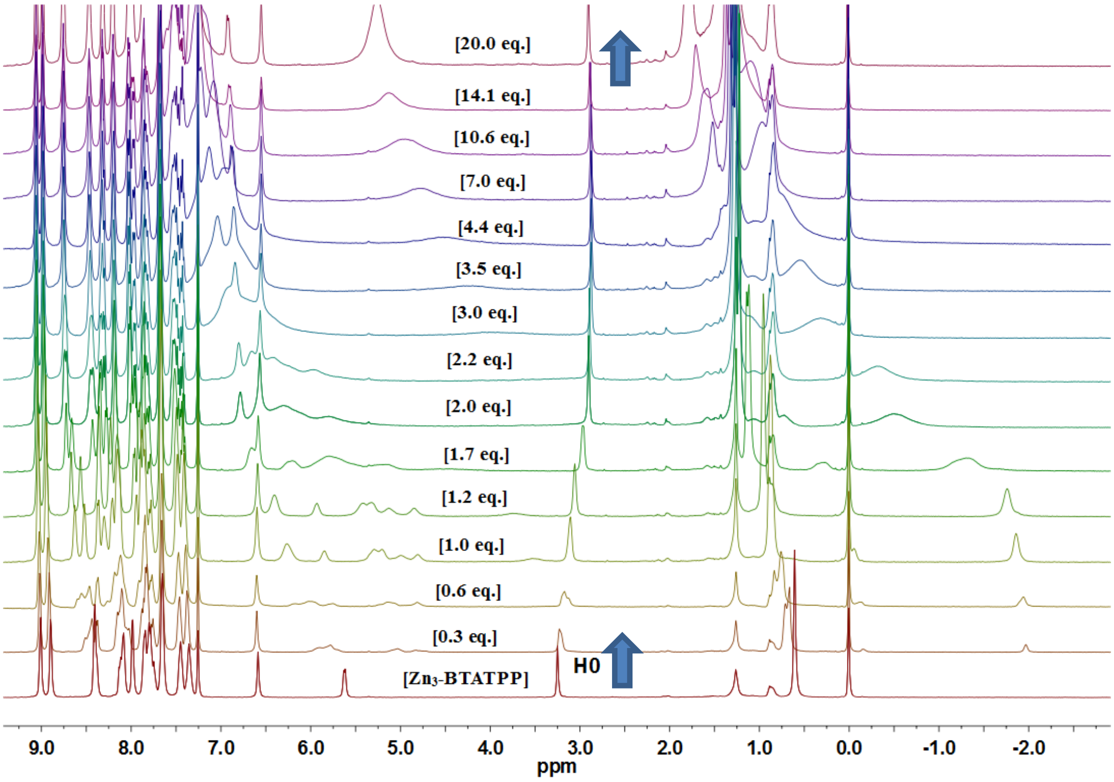

Figure S14. ^1H-NMR spectra of titration of [Zn_3-BTATPP] (4.2×10^{-3} M) with 2R in $CDCl_3$ at 295 K.

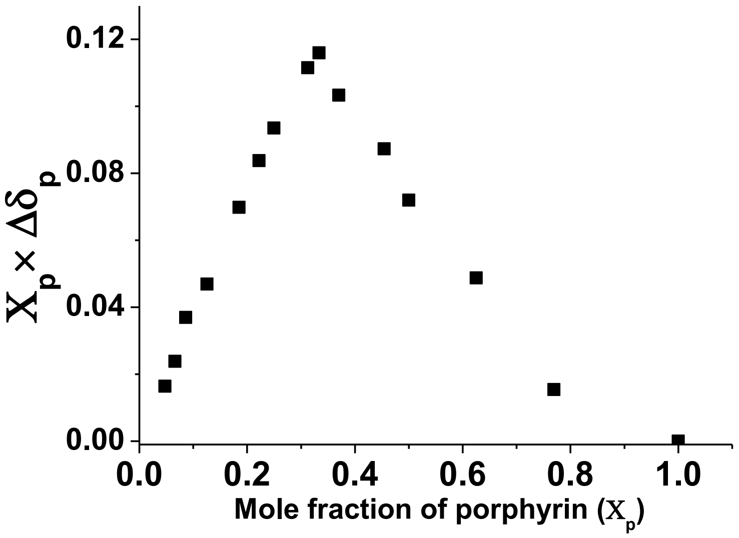

Figure S15. Job's continuous plot of [Zn_3-BTATPP] with 2R (following the changes in chemical shift of [Zn_3-BTATPP] at 3.25 ppm ($\Delta\delta_p$)).

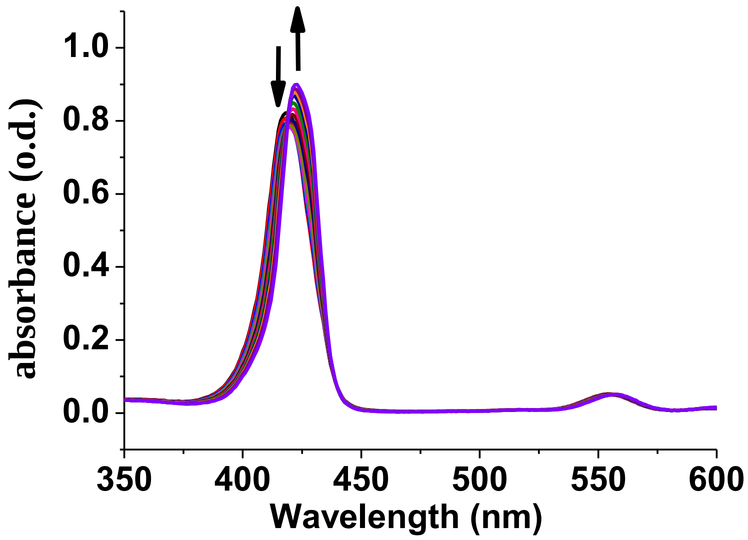

Figure S16. UV-vis spectral change of [Zn_3-BTATPP] (1.2×10^{-6} M) upon addition of 3R as the guest concentrations change from 0-6.6×10^{-2} M.

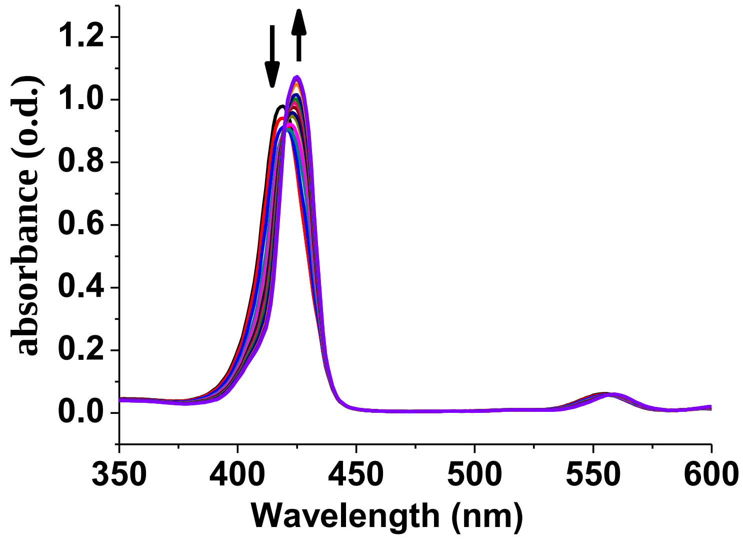

Figure S17. UV-vis spectral change of [Zn_3-BTATPP] (1.3×10^{-6} M) upon addition of 4R as the guest concentrations change from 0-2.7×10^{-2} M.

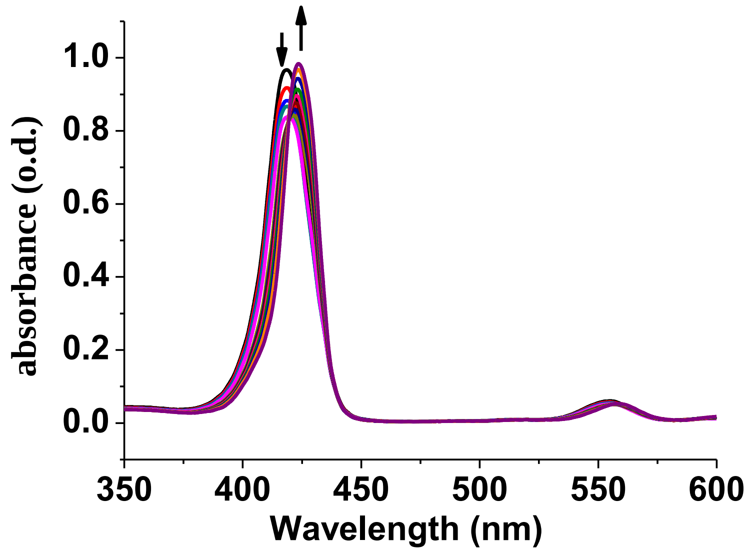

Figure S18. UV-vis spectral change of [Zn_3-BTATPP] (1.3×10^{-6} M) upon addition of 5R as the guest concentrations change from 0-3.5×10^{-2} M.

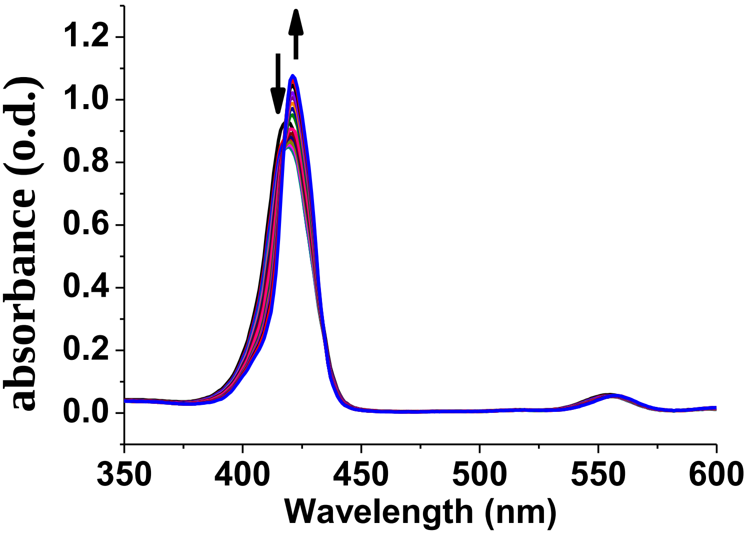

Figure S19. UV-vis spectral change of [Zn_3-BTATPP] (1.3×10^{-6} M) upon addition of 6S as the guest concentrations change from 0-5.5×10^{-2} M.

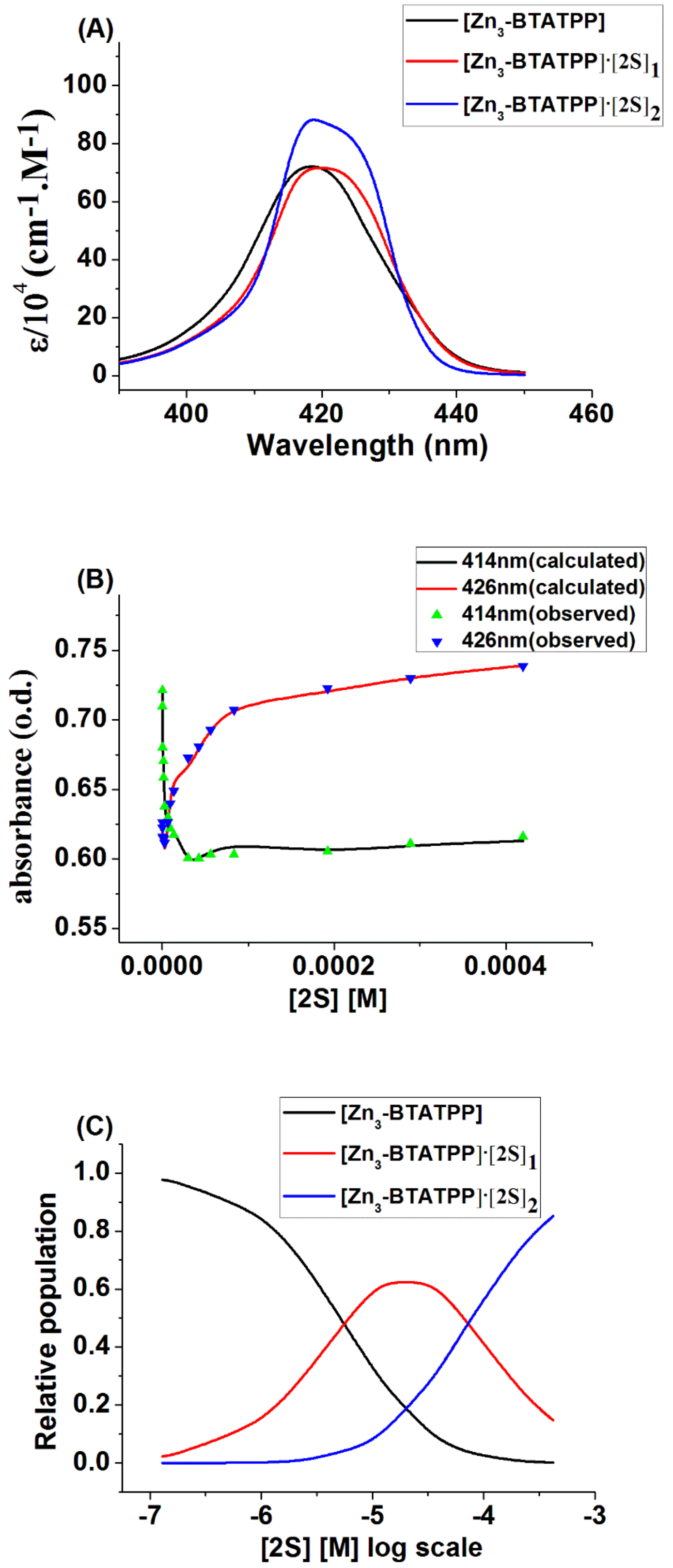

Figure S20. (A) Observed UV-visible spectrum of [Zn$_3$-BTATPP] and calculated UV-visible spectra of [Zn$_3$-BTATPP]·(2S), [Zn$_3$-BTATPP]·(2S)$_2$. (B) Fits of the absorbance data at selected wavelengths of 414 and 426 nm. (C) Species distribution plots of [Zn$_3$-BTATPP]·(2S), [Zn$_3$BTATPP]·(2S)$_2$.

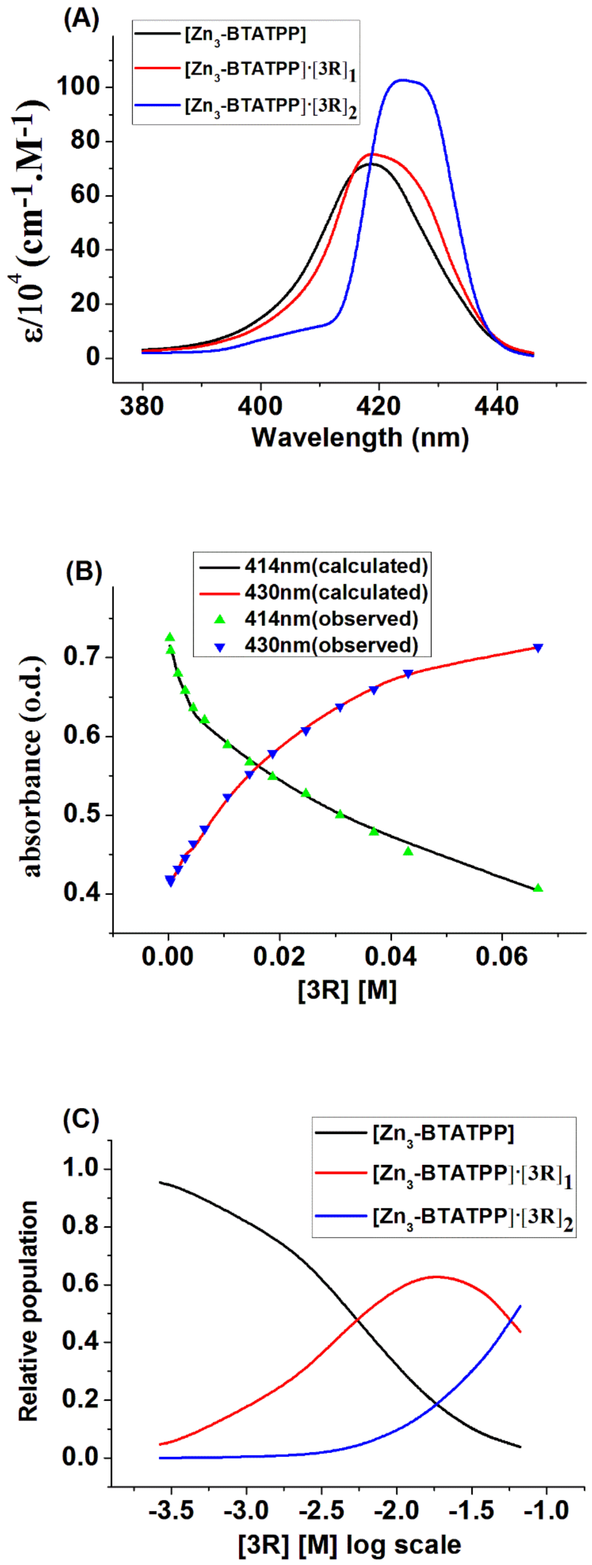

Figure S21. (A) Observed UV-visible spectrum of [Zn_3-BTATPP] and calculated UV-visible spectra of [Zn_3-BTATPP]·(3R), [Zn_3-BTATPP]·$(3R)_2$. (B) Fits of the absorbance data at selected wavelengths of 414 and 430 nm. (C) Species distribution plots of [Zn_3-BTATPP]·(3R), [Zn_3-BTATPP]·$(3R)_2$.

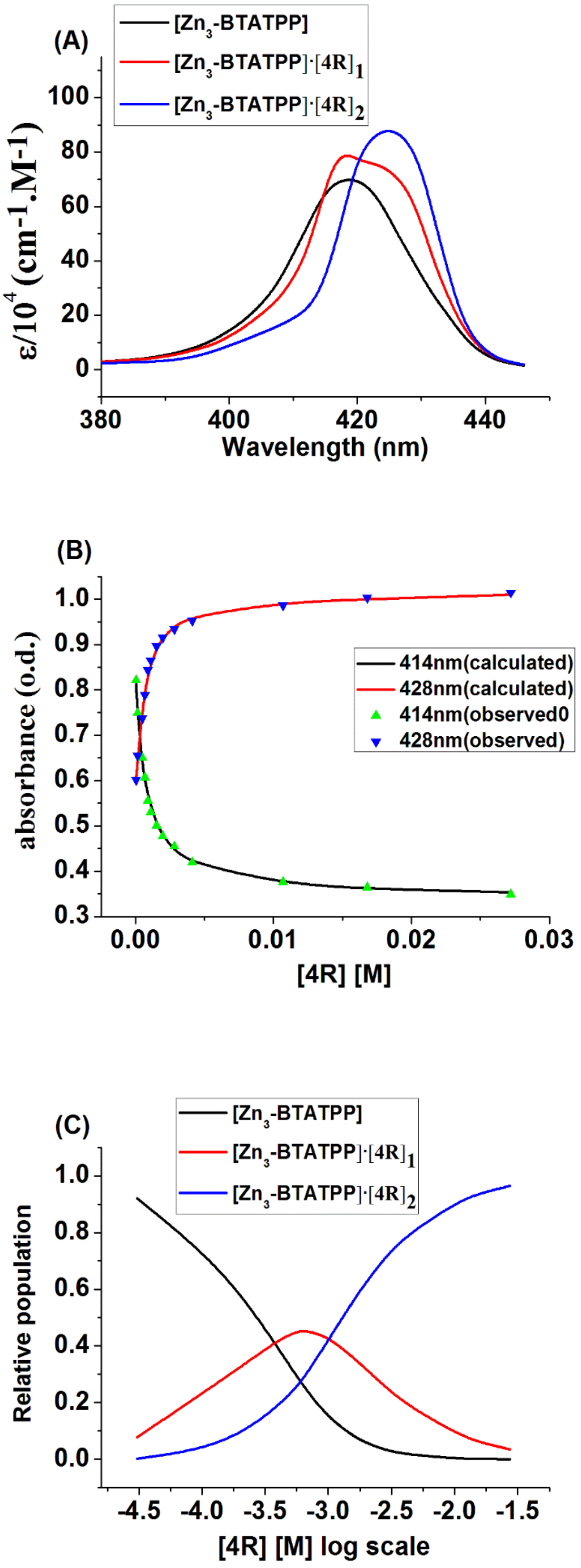

Figure S22. (A) Observed UV-visible spectrum of [Zn_3-BTATPP] and calculated UV-visible spectra of [Zn_3-BTATPP]·(4R), [Zn_3-BTATPP]·$(4R)_2$. (B) Fits of the absorbance data at selected wavelengths of 414 and 428 nm. (C) Species distribution plots of [Zn_3-BTATPP]·(4R), [Zn_3-BTATPP]·$(4R)_2$.

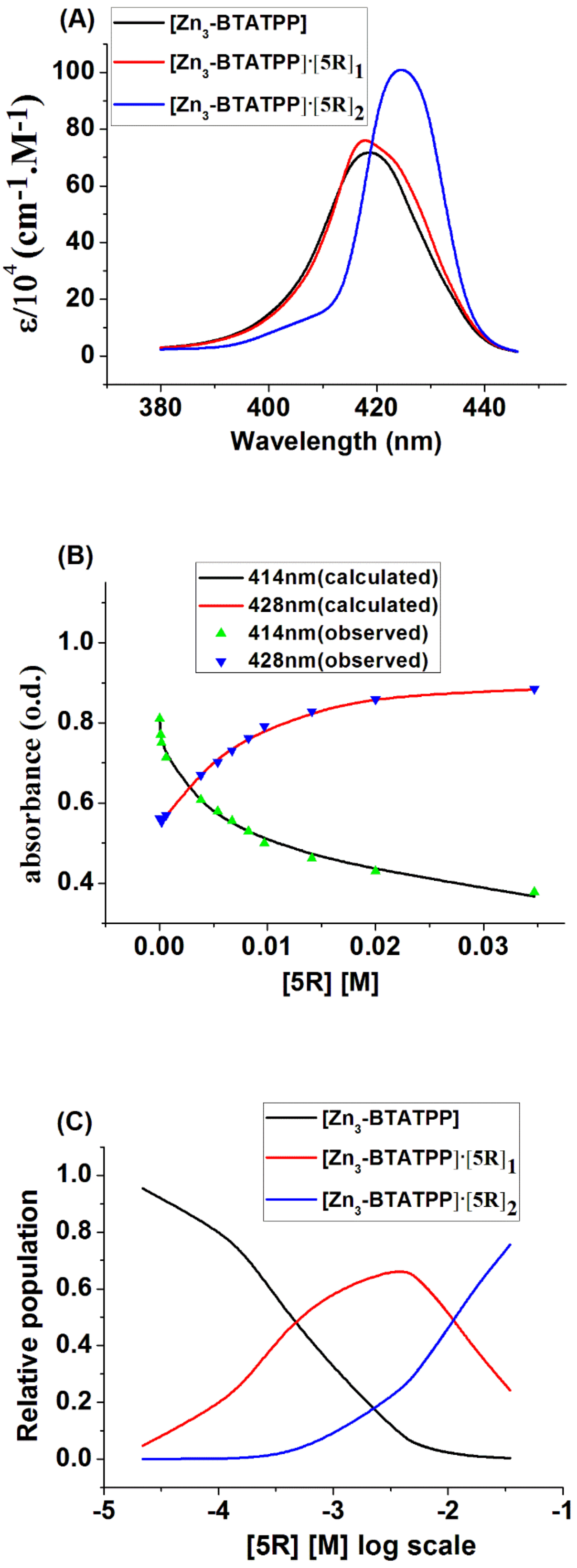

Figure S23. (A) Observed UV-visible spectrum of [Zn_3-BTATPP] and calculated UV-visible spectra of [Zn_3-BTATPP]·(5R), [Zn_3-BTATPP]·$(5R)_2$. (B) Fits of the absorbance data at selected wavelengths of 414 and 428 nm. (C) Species distribution plots of [Zn_3-BTATPP]·(5R), [Zn_3-BTATPP]·$(5R)_2$.

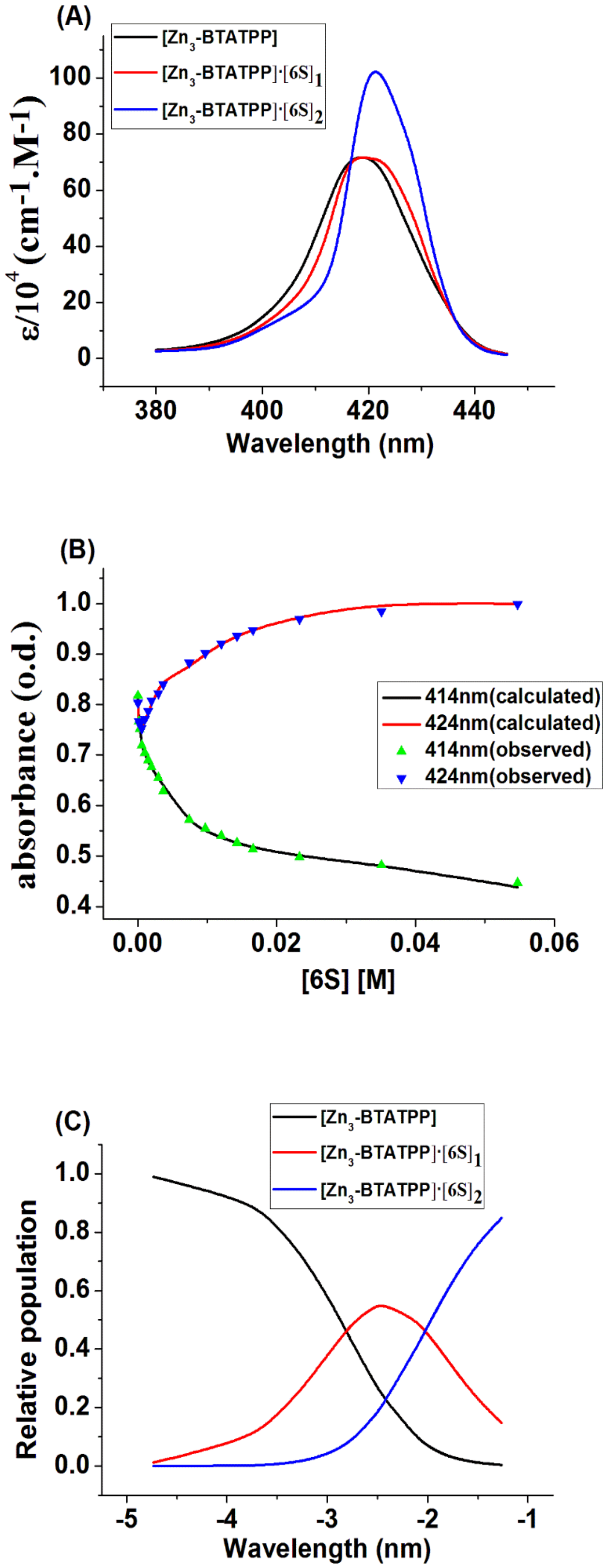

Figure S24. (A) Observed UV-visible spectrum of [Zn_3-BTATPP] and calculated UV-visible spectra of [Zn_3-BTATPP]·(6S), [Zn_3-BTATPP]·$(6S)_2$. (B) Fits of the absorbance data at selected wavelengths of 414 and 424 nm. (C) Species distribution plots of [Zn_3-BTATPP]·(6S), [Zn_3-BTATPP]·$(6S)_2$.

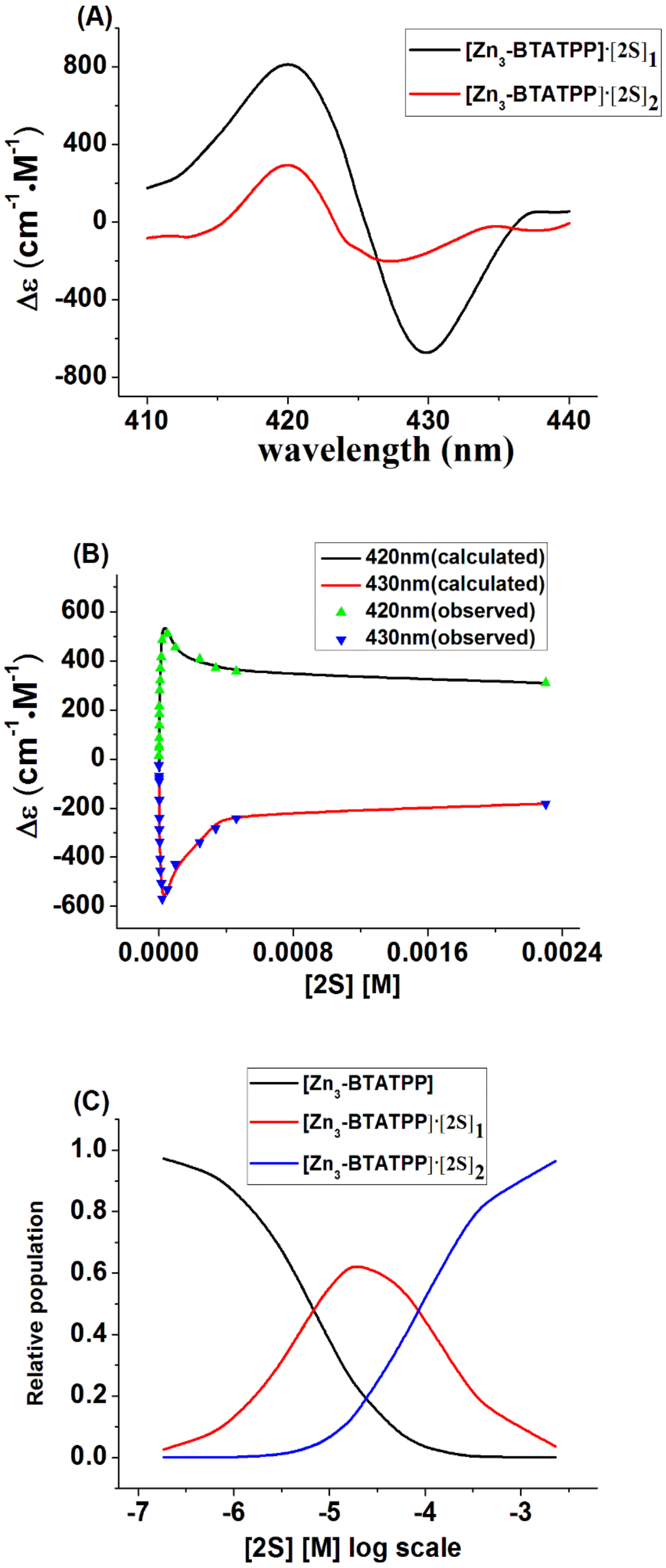

Figure S25. (A) Calculated CD spectra of [Zn_3-BTATPP]•(2S), [Zn_3-BTATPP]•$(2S)_2$. (B) Fits of the CD data at selected wavelengths of 420 and 430 nm. (C) Species distribution plots of [Zn_3-BTATPP], [Zn_3-BTATPP]•(2S), [Zn_3-BTATPP]•$(2S)_2$.

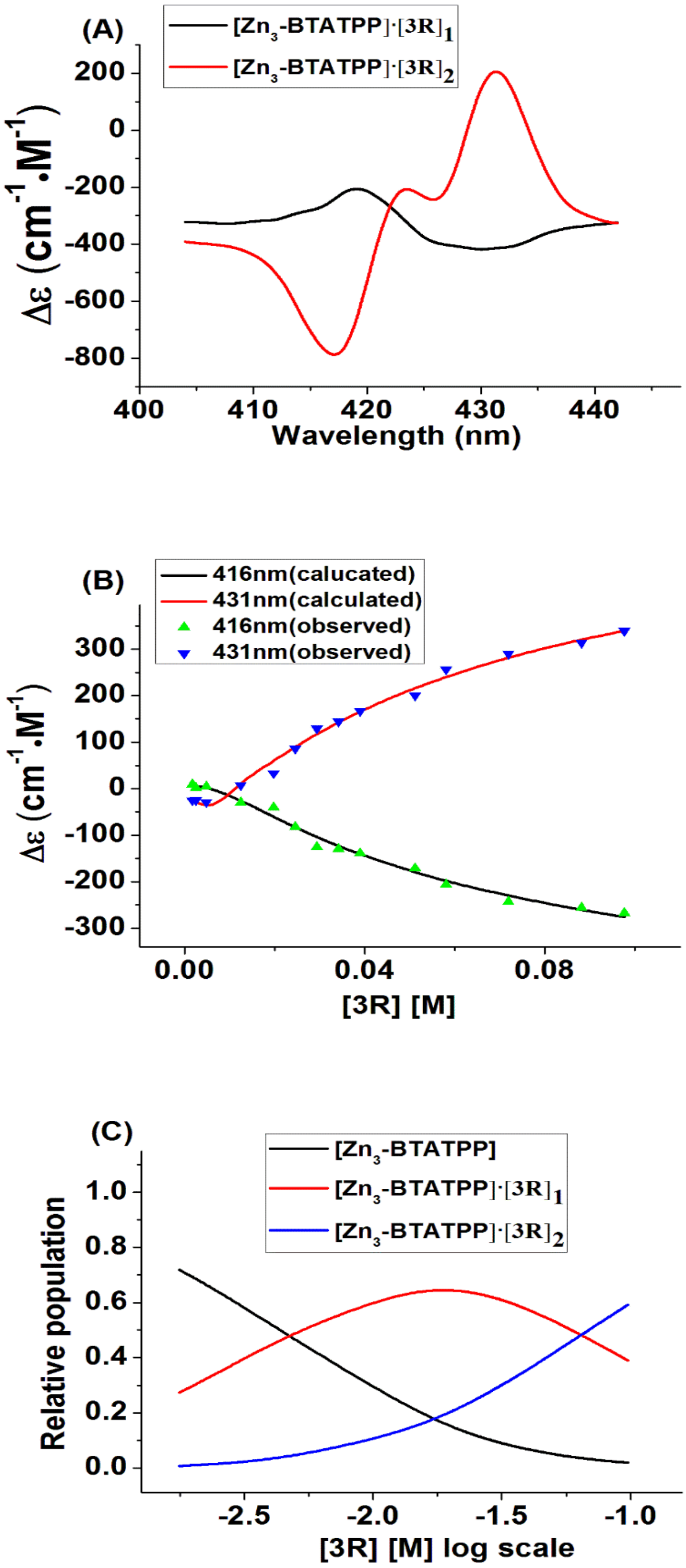

Figure S26. (A) Calculated CD spectra of [Zn_3-BTATPP]•(3R), [Zn_3-BTATPP]•$(3R)_2$. (B) Fits of the CD data at selected wavelengths of 416 and 431 nm. (C) Species distribution plots of [Zn_3-BTATPP], [Zn_3-BTATPP]•(3R), [Zn_3-BTATPP]•$(3R)_2$.

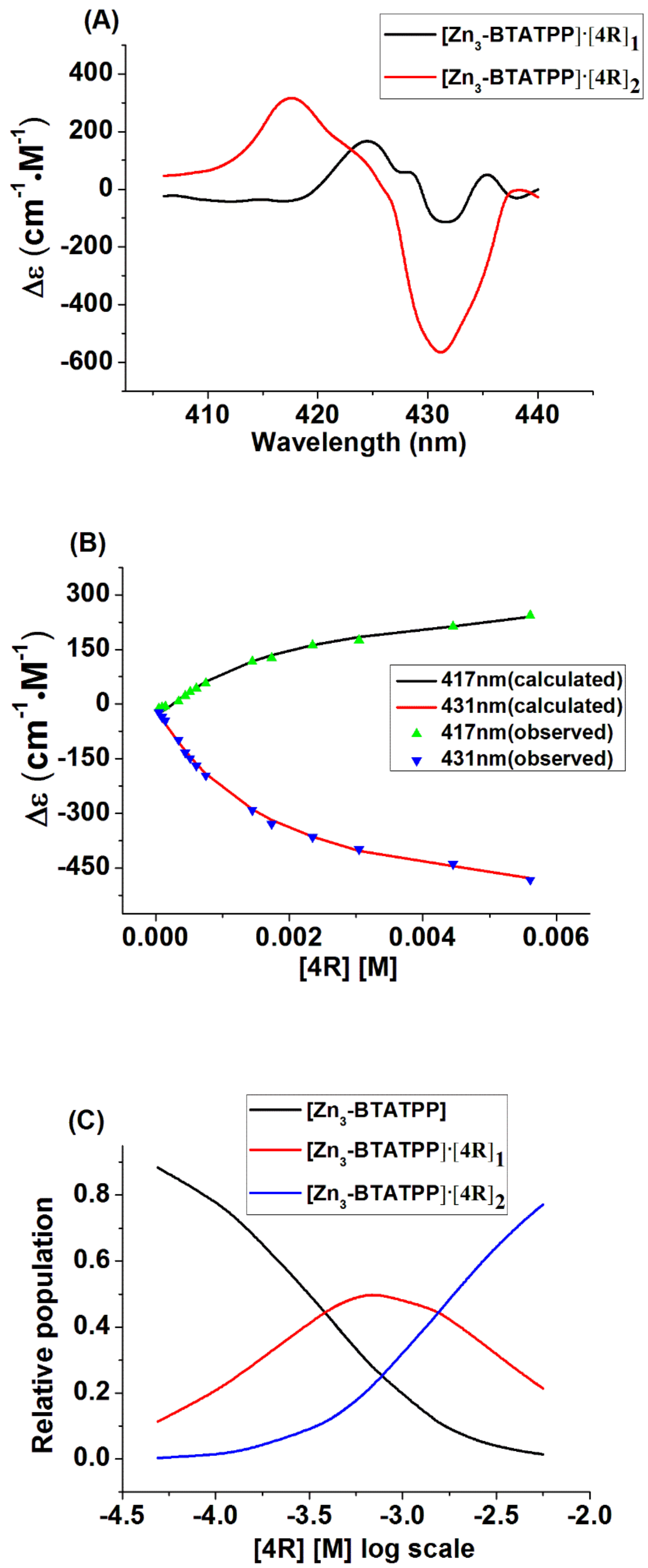

Figure S27. (A) Calculated CD spectra of [Zn_3-BTATPP]•(4R), [Zn_3-BTATPP]•$(4R)_2$. (B) Fits of the CD data at selected wavelengths of 417 and 431 nm. (C) Species distribution plots of [Zn_3-BTATPP], [Zn_3-BTATPP]•(4R), [Zn_3-BTATPP]•$(4R)_2$.

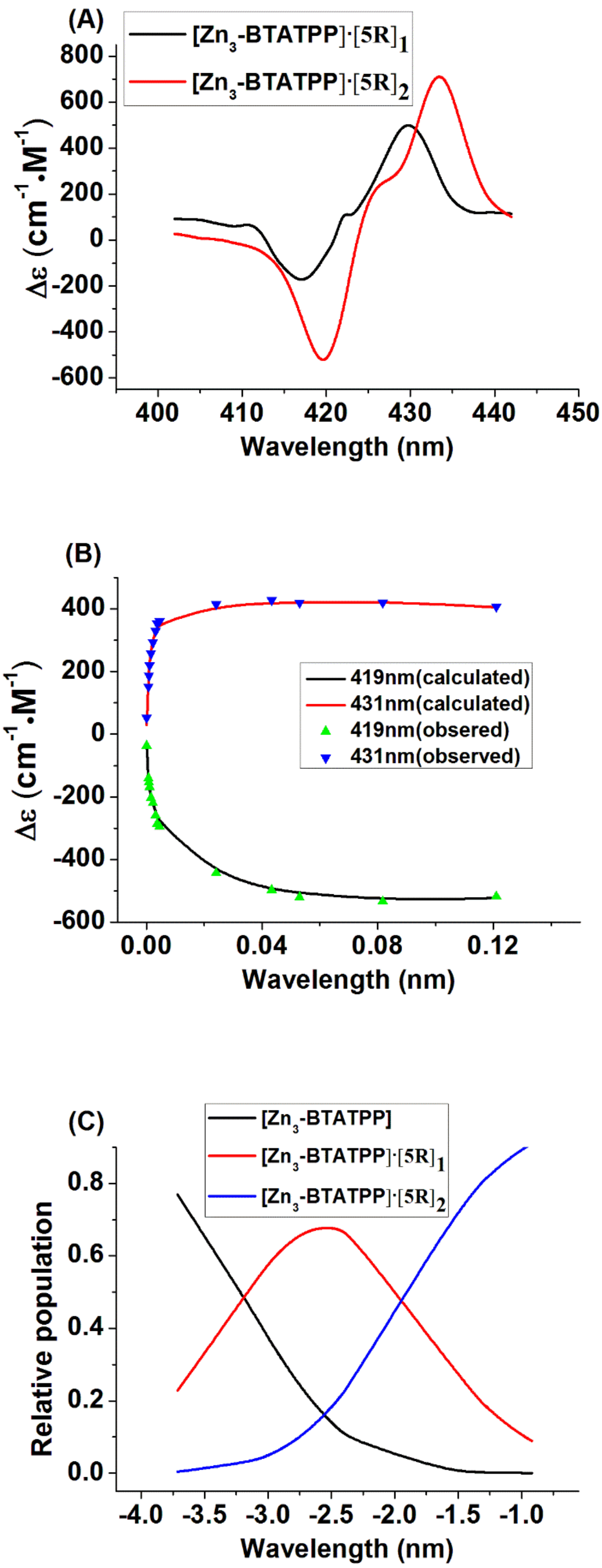

Figure S28. (A) Calculated CD spectra of [Zn_3-BTATPP]•(5R), [Zn_3-BTATPP]•(4R)$_2$. (B) Fits of the CD data at selected wavelengths of 419 and 431 nm. (C) Species distribution plots of [Zn_3-BTATPP], [Zn_3-BTATPP]•(5R), [Zn_3-BTATPP]•(5R)$_2$.

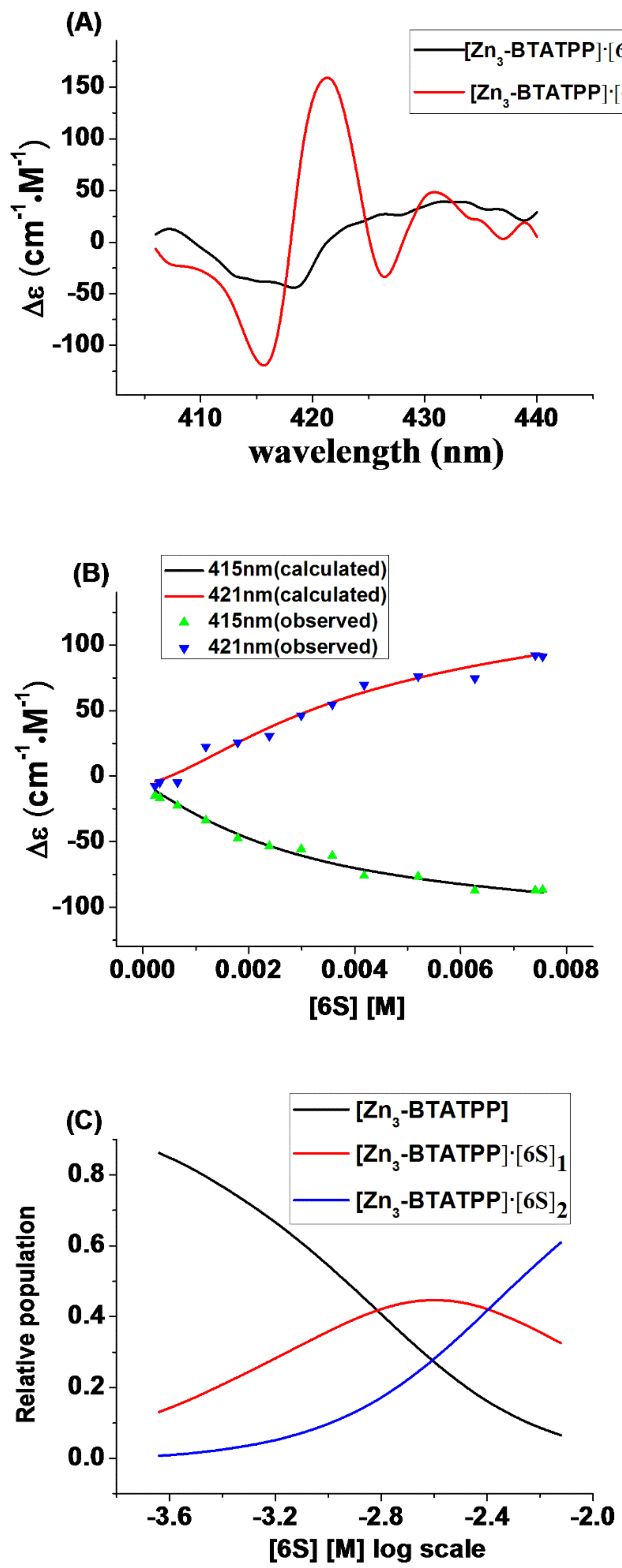

Figure S29. (A) Calculated CD spectra of [Zn_3-BTATPP]•(6S), [Zn_3-BTATPP]•$(6S)_2$. (B) Fits of the CD data at selected wavelengths of 415 and 421 nm. (C) Species distribution plots of [Zn_3-BTATPP], [Zn_3-BTATPP]•(6S), [Zn_3-BTATPP]•$(6S)_2$.

Table S1. Crystal data and structural refinements of $[Zn_3\text{-BTATPP}]\cdot(2S)_2$.

Crystal	$[Zn_3\text{-BTATPP}]\cdot(2S)_2$
Chemical formula	C_{330} H_{222} N_{30} O_{10} Zn_6
Formula weight	5159.58
Wavelength (Å)	0.71073 Å
Temperature	293(2) K
Crystal system	Triclinic
Space group	*P*1
a (Å)	13.299(2)
b (Å)	17.617(3)
c (Å)	30.031(5)
α (deg)	92.208(5)
β(deg)	90.954(5)
γ(deg)	110.756(5)
V ($Å^3$)	6571.0(17)
Z	1
Density (Mg/m^3)	1.304
Absorption coefficient (mm^{-1})	0.609
F(000)	2672
Data collection θ range	2.146-25.00 °
Index ranges	-15<=h<=15<br>-20<=k<=20<br>-35<=l<=35
Reflections collected	310807
R_{int}	0.0674
Independent reflections	45549
Data / restraints / parameters	45549 / 1448 / 2938
GOF on F^2	0.910
$R1^a$ [I> 2σ(I)]	0.0694
wR2	0.1975
Residual peak/hole (e/$Å^3$)	0.917/-0.824

$^aR_1 = (F_0 - F_c)/F_0$, $wR2 = w(F_0^2 - F_c^2)^2/w(F_0^2)\ ^2]^{1/2}$

Table S2. Selected bond distances for [Zn_3-BTATPP]·$(2S)_2$.

Zn(1)-N(11)	2.068(9)	Zn(3)-N(31)	2.040(11)	Zn(5)-N(52)	2.051(11)
Zn(1)-N(12)	2.033(9)	Zn(3)-N(32)	2.019(11)	Zn(5)-N(53)	2.028(13)
Zn(1)-N(13)	2.042(9)	Zn(3)-N(33)	2.038(10)	Zn(5)-N(54)	2.029(12)
Zn(1)-N(14)	2.037(10)	Zn(3)-N(34)	2.069(12)	Zn(5)-O(5)	2.161(11)
Zn(1)-O(1)	2.175 (11)	Zn(4)-N(41)	2.050(10)	Zn(6)-N(61)	2.030(13)
Zn(2)-N(21)	2.053(9)	Zn(4)-N(42)	2.085(11)	Zn(6)-N(62)	2.053(13)
Zn(2)-N(22)	2.090(10)	Zn(4)-N(43)	2.073(11)	Zn(6)-N(63)	2.005 (14)
Zn(2)-N(23)	2.050(9)	Zn(4)-N(44)	2.049(11)	Zn(6)-N(64)	1.967(14)
Zn(2)-N(24)	2.072(9)	Zn(4)-O(4)	2.174(11)		
Zn(2)-O(2)	2.210(8)	Zn(5)-N(51)	2.014(13)		

symmetry code: a) -1+X, -1-Y, 1/2+Z, b) X, -1-Y, 1/2+Z, c) -1+X, -Y, 1/2+Z, d) 1/2+X, 1/2+Y, Z

Table S3. Bond lengths (Å) and angles (°) for hydrogen bonds for [Zn_3-BTATPP]·$(2S)_2$.

D-H...A	D-H	H…A	D…A	＜(DHA)
O1-H1-O013	0.820	1.977	2.673	142.17
O2-H2-O011	0.819	1.930	2.737	168.62
O4-H4-O023	0.821	2.044	2.725	140.09
O5-H5-O021	0.821	1.930	2.750	177.10

Table S4. Analysis of π···π interactions (Å, °).

	Cg-Cg [a]	Alpha[b]	Slippage [c]
ring(1) [d] to ring (5)[h]	3.715	8.36	0.463
ring(2) [e] to ring (5)	3.550	1.99	0.735
ring(3) [f] to ring (6) [i]	3.547	11.09	0.403
ring(4)[g] to ring (6)	3.750	4.84	0.346

[a]Cg-Cg = Distance between ring Centroids; [b]Alpha = Dihedral Angle between Planes I and J; [c]Slippage = Distance between Cg(I) and Perpendicular Projection of Cg(J) on Ring I. Symmetry code: (i) $-x+2$, $-y$, $-z+1$. [d]Ring(1) is composed of C01A, C01B, C01C, C01D, C01E, C01F, C01G, C01H, C01I, C01J; [e]ring(2) is composed of C02A, C02B, C02C, C02D, C02E, C02F, C02G, C02H, C02I, C02J; [f]ring(3) is composed of C03A, C03B, C03C, C03D, C03E, C03F, C03G, C03H, C03I, C03J; [g]ring(4) is composed of C04A, C04B, C04C, C04D, C04E, C04F, C04G, C04H, C04I, C04J; [h]ring(5) is composed of C11, C12, C13, C14, C15, C16; [i]ring(6) is composed of C21, C22, C23, C24, C25, C26.

Table S5. Analysis of C-H···π interactions (Å, °).

	H···Cg[a]	Gamma[b]	C-H···Cg[c]	C···Cg[d]
C01G-H01G to ring(1)[e]	2.81	17.02	144	3.612(9)
C01L-H01E to ring(2)[f]	2.78	21.53	133	3.505(19)
C02I-H02I to ring(3)[g]	2.82	25.19	135	3.538(16)
C02L-H02L to ring(4)[h]	2.80	27.05	128	3.47(2)
C04G-H04G to ring(5)[i]	2.93	12.60	137	3.665(11)
C04L-H04E to ring(6)[j]	2.79	16.22	147	3.63(2)
C05B-H05B to ring(7)[k]	2.79	15.03	165	3.695(13)
C05D-H05D to ring(8)[l]	2.88	13.95	168	3.800(13)
C05J-H05J to ring(9)[m]	2.95	13.02	166	3.854(13)

[a]Cg(J) = Center of gravity of ring J; [b]Gamma = Angle between Cg-H vector and ring J normal; [c]C-H..Cg = C-H-Cg angle; [d]C..Cg = Distance of C to Cg. [e]Ring (1) is composed of N12, C1A1, C1B1, C1B2, C1A2; [f]ring (2) is composed of Zn1, N13, C1A4, C1M3, C1A5, N14; [g]ring (3) is composed of N34, C3A5, C3B5, C3B6, C3A6; [h]ring (4) is composed of Zn2, N22, C2A4, C2M4, C2A5, N23. [i]Ring(5) is composed of N42, C4A1, C4B1, C4B2, C4A2; [j]Ring(6) is composed of N43, C4A3, C4B3, C4B4, C4A4; [k]Ring(7) is composed of N51, C5A1, C5B1, C5B2, C5A2; [l]Ring(8) is composed of N61, C6A7, C6B7, C6B8, C6A8; [m]Ring(9) is composed of Zn6, N61, C6A7, C6M4, C6A6, N64.

Table S6. The calculated binding constants between [Zn_3-BTATPP] and monoalcohols.

	From UV-vis spectra		From CD spectra	
	K_1 and K_2	Standard deviation	K_1 and K_2	Standard deviation
2S	K_1=1.8(±0.1)×10^5<br>K_2=1.4(±0.1)×10^4	3.6×10^{-03}	K_1=1.5(±0.2)×10^5<br>K_2=1.2(±0.1)×10^4	1.0×10^{-02}
3R	K_1=1.8(±0.2)×10^2<br>K_2=1.6(±0.3)×10^1	4.2×10^{-03}	K_1=2.2(±0.2)×10^2<br>K_2=1.6(±0.1)×10^1	3.7×10^{-03}
4R	K_1=2.8(±0.1)×10^3<br>K_2=9.9(±0.8)×10^2	4.5×10^{-03}	K_1=2.6(±0.4)×10^3<br>K_2=6.5(±0.8)×10^2	3.9×10^{-03}
5R	K_1=2.3 (±0.3)×10^3<br>K_2=9.1 (±1.3)×10^1	5.1×10^{-03}	K_1=1.5 (±0.1)×10^3<br>K_2=8.6(±1.3)×10^1	9.8×10^{-03}
6S	K_1=6.5(±0.6)×10^2<br>K_2=1.1(±0.1)×10^2	4.1×10^{-03}	K_1=8.3(±1.7)×10^2<br>K_2=1.8(±0.4)×10^2	1.5×10^{-02}
